# ZO-2 is a scaffold at the centriole and mitotic spindle poles that enhances microtubule stability and supports the proper development of mitotic spindles and cilia

**DOI:** 10.1007/s00441-025-03992-0

**Published:** 2025-07-29

**Authors:** Sara Vega-Torreblanca, Diana Cristina Pinto-Dueñas, Christian Hernández-Guzmán, Dolores Martín-Tapia, Lourdes Alarcón, Bibiana Chávez-Munguía, Lizbeth Salazar-Villatoro, Sirenia González-Pozos, Josué David Hernández-Varela, Leticia Ramírez-Martínez, Esther López-Bayghen, José Jorge Chanona-Pérez, Lorenza González-Mariscal

**Affiliations:** 1 Fisiología, Biofísica y Neurociencias, Centro de Investigación y de Estudios Avanzados del Instituto Politécnico Nacional, Cinvestav, Av. IPN 2508, 07360 CDMX, México; 2 Department of Infectomics and Molecular Pathogenesis, Cinvestav, Av. IPN 2508, 07360 CDMX, México; 3 Electron Microscopy Unit, LaNSE, Cinvestav, Av. IPN 2508, 07360 CDMX, México; 4 Departament of Biochemical Engineering, ENCB, National Polytechnical Institute (IPN), Av. Wilfrido Massieu S/N, CDMX, 07738 México; 5 Department of Toxicology, Cinvestav, Av. IPN 2508, CDMX, 07360 México

**Keywords:** ZO-2, Centrosome, Primary cilium, Mitotic spindle, Spermatozoid

## Abstract

Previous studies revealed the presence of several tight junction (TJ) proteins in the centrosome and their interaction with various centriolar proteins, prompting us to analyze whether this also applies to the TJ protein ZO-2. Here, we found that ZO-2 colocalizes with CEP164 in the distal appendage of the mother centriole and is also present in the pericentriolar region, mitotic spindle poles, the basal body of primary cilia, and the tail of spermatozoa. The absence of ZO-2 altered the cellular content of centriolar proteins CEP164, centriolin, and CEP135, but did not change the morphology of centrioles. ZO-2 depletion inhibits the development of astral and mitotic spindle microtubules expressing EB1. At the spindle poles, ZO-2 depletion increases the accumulation of NuMA while reducing the levels of kinesin KIF14 and the TPX2 scaffold, and the accumulation of the kinase p-Aurora, leading to a decrease in mitotic spindle length, microtubule instability, and abnormal chromosome congression. KIF14, NuMA, and p-Aurora co-immunoprecipitate with ZO-2, and NuMA and Aurora-A bind to different segments of ZO-2. At the ciliary basal body, ZO-2 depletion reduces the content of CEP164, KIF14, and IFT-B protein IFT57, while increasing the expression of p-Aurora and pAKT. These changes block primary cilium development and the response to Sonic Hedgehog signaling pathway stimulation. These results suggest that, rather than being a centrosomal architectural component, ZO-2 enhances microtubule stability and serves as a scaffold that facilitates the adequate accumulation of spindle pole and centriole proteins, allowing proper poleward spindle microtubule flux and cilia development.

**Supplementary Information:**

The online version contains supplementary material available at 10.1007/s00441-025-03992-0.

## Introduction

Tight junctions (TJs) are cell–cell adhesion structures located at the boundary between the apical and basolateral membranes of epithelial cells, regulating the passage of ions and molecules through the paracellular pathway. Using freeze-fracture electron microscopy (Staehelin, et al. 1969) or immunofluorescence with super-resolution microscopy (Gonschior, et al. 2020), TJs appear as a network of strands encircling the cell beneath the apical microvilli. TJs consist of integral proteins such as occludin, claudins, and JAMs, which associate in the cytoplasm with adaptor proteins including cingulin, ZO-1, ZO-2, and ZO-3 [for review, see (Citi, et al. 2024)].

ZO-2 (Zonula occludens*-*2) and ZO-1 are platforms for polymerizing claudins into TJ strands (Umeda, et al. 2006). ZO-2 contains several nuclear localization and exportation signals that allow the protein to move in and out of the nucleus in a cell density-regulated manner (Chamorro, et al. 2009; Gonzalez-Mariscal, et al. 2006; Quiros, et al. 2013). Within the nucleus, ZO-2 is associated with various transcription factors, represses the transcription of cyclin D1 (Huerta, et al. 2007), and targets genes involved in Wnt (Tapia, et al. 2009; Wetzel, et al. 2017) and Hippo signaling pathways (Dominguez-Calderon, et al. 2016; Gallego-Gutierrez, et al. 2021). Additionally, ZO-2 is a bridge connecting the nucleoskeleton composed of lamin B1 to SUN-1, a protein from the LINC complex that spans the inner nuclear membrane and interacts with nesprins −3 and −4. These proteins extend across the outer nuclear membrane and bind through plectin and kinesin to vimentin and microtubule cytoskeleton (Hernandez-Guzman, et al. 2021). Furthermore, the degradation of ZO-2 in the cytoplasm is inhibited by its interaction with 14–3-3 proteins (Amaya, et al. 2019).

ZO-2 functions as a scaffold protein across various cellular locations due to the multiple domains and motifs present in its sequence. As a member of the MAGUK protein family, ZO-2 is characterized by three PDZ domains followed by a supradomain composed of SH3 and guanylate kinase (GuK) modules. This supradomain is anticipated to fold or stretch in response to mechanotension, like the supradomain found in ZO-1 (Spadaro, et al. 2017). The carboxyl segment of ZO-2 includes a proline-rich domain containing an actin-binding region, concluding with the PDZ binding motif TEL. At the plasma membrane, ZO-2 interacts with the tight junction (TJ) proteins occludin, claudins, JAM-A, ZO-1, and cingulin, as well as with the adherens junction (AJ) proteins afadin, β-catenin, and α-catenin (for review, see (Gonzalez-Mariscal, et al. 2019)).

In proximity-dependent biotinylation (BioID) assays, ZO-2 was found to associate in vivo with several centrosomal proteins, including centriolin, CEP128, and CEP135 (Gupta, et al. 2015), with proteins IFT57 and IFT74 involved in intraflagellar transport in the primary cilium (Go, et al. 2021), with the transition fiber protein FBF-1 that associates with the IFT-B complex (Gupta, et al. 2015), with KIF14 a kinesin present in the ciliary axoneme (Brown, et al. 2015; Go, et al. 2021), and with ODF2 (Outer dense fiber 2) a protein present in the centrosome and the spermatozoid tail (Gupta, et al. 2015). These observations prompted us to explore the interaction of ZO-2 with the centrosome, primary cilium, and spermatozoid tail in this study.

The centrosome is a cellular organelle whose physiology was first studied in the nineteenth century (Scheer 2014). It serves as the main microtubule-organizing center and plays a crucial role in mitotic spindle formation and orientation in cycling cells (Chavali, et al. 2015; Soung, et al. 2009). In contrast, in non-proliferating cells, the centrosome nucleates the microtubule array and functions as a platform for forming primary cilia (Graser, et al. 2007). Additionally, the centrosome is involved in cell polarity determination (Bienkowska and Cowan 2012). Centrosomes consist of two centrioles arranged perpendicularly and surrounded by pericentriolar material. The older of the pair is referred to as the mother centriole (MC), while the other is known as the daughter centriole (DC) [for review, see (Conduit, et al. 2015)]. The MC can be differentiated from the DC because it is surrounded by proteinaceous accessory structures known as the distal appendage (DA) and the subdistal appendage (SDA) (Kashihara, et al. 2019). The DA is composed of the proteins ODF2, CEP83, CEP89, SCLT1, CEP164, and FBf1, which accumulate hierarchically (Graser, et al. 2007; Tanos, et al. 2013). The SDA anchors the microtubules during interphase and integrates with other proteins, including cenexin, CEP128, centriolin, Ndel1, ninein, and CEP170 (Hung, et al. 2016).

Cells that exit the cell cycle and enter quiescence develop primary cilia, antenna-like structures that sense extracellular stimuli and respond to mechanical and chemical cues. The primary cilium consists of a microtubule-based axoneme surrounded by a ciliary membrane, a transition zone at the ciliary base where proteins are targeted and sorted to and from the cilia, and a basal body that derives from the MC and is anchored to the plasma membrane through its DAs, which are now transformed into transition fibers (Reiter, et al. 2012). In ciliogenesis, the transition from the MC to the basal body involves the removal of CP110 and CEP97 from the distal end of the centriole. This process requires TTBK2 activity, which is recruited to the basal body by CEP164 (Cajanek and Nigg 2014).

This study, conducted in epithelial MDCK cells, employs Western blotting, confocal microscopy, and SIM (structured illumination microscopy) super-resolution microscopy to examine whether ZO-2 colocalizes with proteins found in various regions of the centriole. It also investigates the effects of ZO-2 depletion on the abundance and distribution of centriolar proteins. In proliferating cells, it analyzes the influence of ZO-2 silencing on the length of the mitotic spindle and the accumulation of various proteins at the mitotic spindle poles. In addition, ZO-2 immunoprecipitation and pull-down assays were used to explore the interaction of ZO-2 with proteins of the spindle pole. In quiescent cells, the effect of ZO-2 depletion on the concentration of several proteins at the ciliary basal body and the formation of the primary cilium was also studied. Finally, the distribution of ZO-2 in the spermatozoid tail was explored.

## Material and methods

### Cell culture

Parental (control) and ZO-2 KD MDCK cells were kindly provided by Alan Fanning (University of North Carolina, Chapel Hill, NC) and cultured as previously described (Van Itallie, et al. 2009). ZO-2 KD cells stably expressed a specific shRNA against ZO-2 in the pSuper vector, whereas parental cells expressed the empty vector. Clones of ZO-2 KD cells were isolated based on their resistance to zeocin. This work was done with clone IC5, which was previously studied (Raya-Sandino, et al. 2017). Supplemental Fig. 1 confirms the absence of ZO-2 expression in ZO-2 KD cells through immunofluorescence and Western blot analyses. Monolayers were incubated with rabbit pre-immune serum instead of the rabbit anti-ZO-2 antibody as a negative control in the immunofluorescence experiment. According to experimental requirements, cells were plated at confluent (1.5 X 10^5^ cells/cm^2^) or sparse (5 X 10^4^ cells/cm^2^) densities.

To obtain MDCK cells in different stages of the cell cycle, the following procedures were followed according to previous observations (Tapia et al. 2009): 1) G1: 24 h after plating the monolayers at sparse density in DMEM with 10% fetal bovine serum (FBS), the cells were transferred to DMEM with 0.1% FBS for 48 h. 2) S: after 48 h in DMEM with 0.1% FBS, the monolayers were incubated for 12 h in DMEM with 10% FBS. 3) G2/M: sparse unsynchronized cultures were treated for 16 h with 0.3 μM nocodazole, which arrests the cell cycle at the beginning of mitosis by blocking spindle microtubule assembly. 4) Cells in different phases of mitosis: sparse unsynchronized cultures were incubated for 24 h in DMEM with 10% FBS.

For the cell wounding assay, parental and ZO-2 KD cells were plated at confluent density on a glass coverslip. Twenty-four hours later, the monolayers were scratched with the tip of a pipette and incubated at 37 °C. After six hours, the monolayers were fixed and processed for immunofluorescence.

To induce cilia development in parental and ZO-2 KD MDCK cells, which were treated or not treated with 10 μM docetaxel, cells were plated at confluence density on glass coverslips and starved in a serum-depleted medium (0.1% v/v FBS) for 48 h. Monolayers were then fixed and processed for immunofluorescence.

### Western blots

For the Western blots, rabbit polyclonal antibodies were used against ZO-2 (Cat.71–1400, dilution 1:1000, Invitrogen, Rockford, IL), CEP135 (Cat. A302-250A, dilution 1:1,000, Bethyl Laboratories, Montgomery, TX), GAPDH (Cat. RPCA-GAPDH, dilution 1:50,000, Encore Biotechnology Inc., Gainesville, FL), NuMA (Cat. GTX113510, dilution 1:750, GeneTex, Irvine, CA), and 6 × His tag (Cat. GTX115045, dilution 1:5000; Genetex, Irvine, CA, USA). Additionally, a rabbit monoclonal antibody was used against phospho-Aurora A (Thr288)/Aurora B (Thr232)/Aurora C (Thr198)(D13A11)XP (Cat. 2914, dilution 1:1,500, Cell Signaling Technology, Danvers, MA), or mouse monoclonal antibodies were used against centriolin (Cat. sc-365521, dilution 1:200, Santa Cruz Biotechnology, Dallas, TX) and CEP164 (Cat. sc-515403, dilution 1:250, Santa Cruz Biotechnology, Dallas, TX). We also employed the IgG_1_ kappa light chain of mouse monoclonal antibodies against: TPX2 (Cat. sc-390183, dilution 1:250, Santa Cruz Biotechnology, Dallas, TX), IFT57 (Cat. sc-390120, dilution 1:250, Santa Cruz Biotechnology, Dallas, TX), KIF14 (Cat. sc-365553, dilution 1:250, Santa Cruz Biotechnology, Dallas, TX) and Aurora-A (Cat.Sc-373856, dilution 1:500, Santa Cruz Biotechnology, Dallas, TX). As secondary antibodies, peroxidase-conjugated goat antibodies were employed against rabbit IgG (Cat. 62–6120, dilution 1:20,000, Invitrogen, Camarillo, CA) or mouse IgG (Cat. 62–6520, dilution 1:10,000, Invitrogen, Camarillo, CA). For the detection of the IgG_1_ kappa light chain of mouse monoclonal antibodies, we employed mouse IgGκ light chain binding protein conjugated to horseradish peroxidase (m-IgGκ BP-HRP, Cat. sc-516102, dilution 1:250, Santa Cruz Biotechnology, Dallas, TX). We then employed the chemiluminescence detection system, Immobilon™ western (Merck Millipore, Cat. WBKLS 0500, Darmstadt, Germany). Western blots were performed according to standard procedures, as previously reported (Quiros, et al. 2013). For the detection of phospho-Aurora A, the membranes were incubated overnight at 4 °C, under gentle shaking, with the primary antibody diluted in a buffer containing 5% w/v BSA, 1X TBS, and 0.1% Tween 20, as indicated by the antibody manufacturer.

### Immunofluorescence

For the immunofluorescence experiments, the monolayer fixation, permeabilization, and blockade protocols varied based on the antibodies employed, as indicated in Table 1. All monolayers were incubated overnight at 4 °C with the primary antibody diluted in the blocking solution, washed three times with PBS, and incubated at room temperature for 2 h with the secondary antibody diluted in the blocking solution. The secondary antibodies employed were: donkey anti rat IgG coupled to Alexa-Fluor488 (Cat. A-21208, dilution 1:750, ThermoFisher, Waltham, MA), or anti mouse coupled to Alexa-Fluor488 (Cat. A-21202, dilution 1:750, ThermoFisher, Waltham, MA), as well as anti mouse coupled to Alexa-Fluor594 (Cat. A-21203, dilution 1:750, ThermoFisher, Waltham, MA), anti rabbit coupled to Alexa-Fluor488 (Cat. A-21206, dilution 1:750, ThermoFisher, Waltham, MA), and anti rabbit coupled to Alexa-Fluor594 (Cat. A-21207, dilution 1:750, ThermoFisher, Waltham, MA). To detect the mouse IgGκ light chain we employed a mouse IgGκ light chain binding protein coupled to FITC (m-IgGκ BP-FITC, Cat. sc-516140, dilution 1:20, Santa Cruz Biotechnology, Dallas, TX). After three washes with PBS, the monolayers were mounted with Vectashield/DAPI (Cat. No. H-1200, Vector Laboratories Inc., Burlingame, CA).

**Table 1:** Fixation, permeabilization and blockade protocols employed for inmunofluorescence experiments using diverse primary antibodies

**Antibody**		**Fixation**	**Permeabilization**	**Blockade**
**Rabbit α-ZO-2** (Dil. 1:100) BiCell Scientific Cat. 00238 St Louis, MO.		PFA 2% (v/v), 10 min, RT	Triton X-100 0.25% (v/v), 10 min	BSA 0.5% (v/v), 30 min
**Mouse α- α-tubulin** (Dil. 1:100) Sigma Aldrich Cat. T9026 St Louis, MO.		Methanol 100% (v/v), 20 min, –20°C		
**Mouse α-ace-tubulin** (Dil. 1:100) Santa Cruz Biotechnology Cat. sc-23950 Dallas, TX.				
**Rabbit α-Ɣ-tubulin** (Dil. 1:100) GeneTex Cat. GTX113286 Irvine, CA.	**Mouse α-CEP164** (Dil. 1:100) Santa Cruz Biotechnology Cat. sc-515403 Dallas, TX.			
**Rabbit α-Ɣ-tubulin** (Dil. 1:100) GeneTex Cat. GTX113286 Irvine, CA.	**Mouse α-centriolin** (Dil. 1:100) Santa Cruz Biotechnology Cat. sc-365521 Dallas, TX.			
**Mouse α-Ɣ-tubulin** (Dil. 1:100) Santa Cruz Biotechnology Cat. sc-17787 Dallas, TX.	**Rabbit α-CEP135** (Dil. 1:100) Bethyl Laboratories Cat. A302-250A Montgomery, TX.			
**Rabbit α-ZO-2** (Dil. 1:100) BiCell Scientific Cat. 00238 St Louis, MO.	**Rat α-CEP135** (Dil. 1:100) BiCell Scientific Cat. KIAA0635 St Louis, MO.	PFA 2% (v/v), 10 min, RT	Triton X-100 0.25% (v/v), 10 min	
**Rabbit α-ZO-2** (Dil. 1:100) Invitrogen Cat. 71-1400 Rockford, Il.	**Mouse α-Ɣ- tubulin** (Dil. 1:100) Santa Cruz Biotechnology Cat. sc-17787 Dallas, TX.			
**Rabbit α-ZO-2** (Dil. 1:100) Invitrogen Cat. 71-1400 Rockford, Il.	**Mouse α-CEP164** (Dil. 1:100) Santa Cruz Biotechnology Cat. sc-515403 Dallas, TX.			
**Rabbit α-ZO-2** (Dil. 1:100) Invitrogen Cat. 71-1400 Rockford, Il.	**Mouse α-centriolin** (Dil. 1:100) Santa Cruz Biotechnology Cat. sc-365521 Dallas, TX.			
**Rabbit α-ZO-2** (Dil. 1:100) Invitrogen Cat. 71-1400 Rockford, Il.	**Mouse α-TPX2** (Dil. 1:20) Santa Cruz Biotechnology Cat. sc-390183 Dallas, TX.			
**Rabbit α-ZO-2** (Dil. 1:100) Invitrogen Cat. 71-1400 Rockford, Il.	**Mouse α-ace-tubulin** (Dil. 1:100) Santa Cruz Biotechnology Cat. sc-23950 Dallas, TX.	PFA 2% (v/v), 10 min, RT	Triton X-100 0.25% (v/v), 10 min	BSA 0.5% (v/v), 30 min
**Rabbit α-NuMA** (Dil. 1:100) GeneTex Cat. GTX113510 Irvine, CA.	**Mouse α-Ɣ-tubulin** (Dil. 1:100) Santa Cruz Biotechnology Cat. sc-17787 Dallas, TX	Methanol 100% (v/v), 20 min, –20°C		
**Mouse α-TPX2** (Dil. 1:20) Santa Cruz Biotechnology Cat. sc-390183 Dallas, TX.	**Rabbit α-ace-tubulin** (Dil. 1:100) Cell Signaling Cat. 5335 Danvers, MA.			
**Mouse α-KIF14** (Dil. 1:20) Santa Cruz Biotechnology Cat. sc-365553 Dallas, TX.	**Mouse α-ace-tubulin** (Dil. 1:100) Santa Cruz Biotechnology Cat. sc-23950 Dallas, TX.			
**Rabbit α-p-Aurora** (Dil. 1:100) Cell Signaling Cat. 2914 Danvers, MA.	**Mouse α-ace-tubulin** (Dil. 1:100) Santa Cruz Biotechnology Cat. sc-23950 Dallas, TX.			
**Rabbit α-ace-tubulin** (Dil. 1:100) Cell Signaling Cat. 5335 Danvers, MA.	**Mouse α-CEP164** (Dil. 1:100) Santa Cruz Biotechnology Cat. sc-515403 Dallas, TX.			
**Rabbit α-p-AKT** (Dil. 1:100) Cell Signaling Cat. 2914 Greenbelt, MD	**Mouse α-ace-tubulin** (Dil. 1:100) Santa Cruz Biotechnology Cat. sc-23950 Dallas, TX.			
**Rabbit α-p-Aurora** (Dil. 1:100) Cell Signaling Cat. 2914 Danvers, MA.	**Mouse α-Ɣ-tubulin** (Dil. 1:100) Santa Cruz Biotechnology Cat. sc-17787 Dallas, TX			
**Mouse α-ace-tubulin** (Dil. 1:100) Santa Cruz Biotechnology Cat. sc-23950 Dallas, TX.	**Rabbit α-Ɣ-tubulin** (Dil. 1:100) GeneTex Cat. GTX113286 Irvine, CA.			
**Mouse α-EB1** (Dil. 1:100) Santa Cruz Biotechnology Cat. sc-47704 Dallas, TX.	**Rabbit α-Ɣ-tubulin** (Dil. 1:100) GeneTex Cat. GTX113286 Irvine, CA.			
**Rat α-ZO-1** (Dil. 1:10) DSHB Cat. R26.4C University of Iowa, IA	**Mouse α-ace-tubulin** (Dil. 1:100) Santa Cruz Biotechnology Cat. sc-23950 Dallas, TX.	Methanol 100% (v/v), 20 min, –20°C		BSA 0.5% (v/v), 30 min
**Mouse α-IFT57** (Dil. 1:20) Santa Cruz Biotechnology Cat. sc- 390120 Dallas, TX	**Mouse α-ace-tubulin** (Dil. 1:100) Santa Cruz Biotechnology Cat. sc-23950 Dallas, TX.			

### Confocal imaging

The samples were observed using confocal laser scanning microscopy (CLSM, TCS-SP8 DM6000, Leica, Germany, or TCS-SPE DM400, first generation, Leica, Germany) with laser excitation diodes ranging from 405 to 633 nm. ImageJ software version 1.53 s (National Institutes of Health, USA) was employed for image analysis.

### Super-resolution imaging (SIM)

Immunofluorescence samples previously observed using confocal microscopy were evaluated with a 100 × 1.46 Zeiss a-Plan Apochromat oil-immersion objective DIC M27 in an inverted confocal laser scanning microscope with super-resolution settings (LSM 880-Elyra PS.1, Carl Zeiss, Germany). The SIM images were captured using the z-stack function, allowing for the reconstruction of 36 images (z = 5.25 µm) with three-phase image rotation. Gratings of 51 µm (488 nm) and 34 μm (561 nm) were used for each laser. An area of 256 × 256 pixels (16.6 µm) was selected for study, with a frame exposure time of 100 ms. The ImageJ software version 1.53 s (National Institutes of Health, USA) was utilized for image analysis.

### RT-qPCR

Parental or ZO-2 KD MDCK cells were seeded into 24-well plates. For the detection of CEP164 and centriolin mRNA, confluent monolayers of parental and ZO-2 KD cells were employed. Instead, to detect Gli1, the monolayers were transferred 24 h after being plated in CDMEM to serum-free media with 1.6 μM of smoothened agonist SAG. After 24 h the monolayers were harvested. Total RNA was isolated for both experiments using the Direct-zol MiniPrep kit (Cat. R2050; Zymo Research, Irvine, CA). RT-qPCR was performed with the KAPA SYBR FAST One-Step qRT-PCR Kit (Cat. KK4650; Kapa Biosystems, Basilea, Switzerland) in a reaction volume of 10 ml [20 ng of total RNA, Rox reference dye (1X), Kapa RT mix (1X) Kappa SYBR master mix (1X), forward primer (10 nM) and reverse primer (10 nM)]. Gene expression was measured using the ABI Step One Plus Real-Time PCR System (Cat. 4,376,600; Applied Biosystems, Carlsbad, CA). The reaction consisted of a cDNA synthesis step at 42 °C for 5 min, followed by inactivation of the RT at 95 °C for 5 min, and then 40 cycles at 95 °C for 3 s and finally at 60 °C for 30 s. Melting curves were constructed to determine the product’s purity and verify its identity. The relative abundance of each mRNA was expressed as sample versus control compared to *PRP0* mRNA, which was calculated using the 2^−ΔΔCt^ method. For primer sequences, see Table 2.

**Table 2:** Specific primers sequences

Gene	Primer	Sequence
*centriolin*	Forward	5´ TCTTTGCGAATCCTGAATCTG
	Reverse	5´ CTACCTCATCTAAACTGAATCTCG
*CEP164*	Forward	5´ GAGTGACAATCAGAGTGTCCG
	Reverse	5´ CTGGTTGGCTTGTTCTTGGAG
*PRP0*	Forward	5´TACAACCCTGAAGTGCTTGAC
	Reverse	5´GCAGATGGATCAGCCAAGAAG
*Gli1*	Forward	5´ ACTTGCCAGCTAAAGTCTGAG
	Reverse	5´ CGTCTCATAGGCAGATTCGG

### Immunoprecipitation

Following a previously described protocol (Raya-Sandino, et al. 2017), ZO-2 was immunoprecipitated using 1 μl of antibody against ZO-2 (Cat. 71–1400, Invitrogen, Carlsbad, CA) per 300 μg of protein in the lysate of parental MDCK cells. The radioimmunoprecipitation assay buffer employed contained 50 mM Tris–HCl, pH 7.5, 150 mM NaCl, 1% NP-40 (v/v), and the protease inhibitor cocktail Complete™ (Cat. 11,697,498,001, Roche Diagnostics, Mannheim, Germany).

### Obtention of mice and human spermatozoids

Adult male ICR-CD1 mice at 12 weeks of age were obtained from the Cinvestav animal facility and maintained under 12-h dark–light cycles, with food and water available ad libitum. Animals were sacrificed via cervical dislocation. Sperm were collected from the caput epididymis to the *vas deferens* by injecting PBS with a 30-gauge needle. The flushed-out spermatozoa were washed three times with PBS at 3,000 rpm for 5 min. The spermatozoa were then fixed with 3% (v/v) paraformaldehyde for 10 min at room temperature, washed with cold PBS, and centrifuged for 1 min at 3,000 rpm. Samples were permeabilized with 0.25% (v/v) Triton X-100 for 10 min at room temperature, washed with cold PBS, and again centrifuged for 1 min at 3,000 rpm. Subsequently, the cells were smeared onto coverslips, left for 10 min to dry, blocked with 1% (w/v) BSA for 1 h at room temperature, and processed for immunofluorescence. All animal procedures were conducted in accordance with international regulations, Mexican official norm NOM-062-ZOO-1999, and the Institutional Animal Care and Use Committee (CICUAL, protocol 29–13).

The human sperm sample was collected by masturbation from a normozoospermic donor. The samples were washed three times with PBS at 3,000 rpm for 5 min. A written informed consent was obtained (signed) from all participants whose samples were included in the study. Consent for publication was not applicable.

### Transmission electron microscopy

Parental and ZO-2 KD MDCK monolayers were fixed with 2.5% (v/v) glutaraldehyde (Cat. 16,365, Hatfield, PA) in 0.1 M sodium cacodylate buffer (Cat. C0250, SIGMA, St. Louis, MO), pH 7.2, for 60 min at room temperature. They were then postfixed for 60 min with 1% (w/v) osmium tetroxide (Cat. 19,110, Hatfield, PA) in the same buffer. After dehydration with increasing concentrations of ethanol and propylene oxide, samples were embedded in Poly/bed 812 resin (Polysciences Inc., Warrington, PA) and polymerized at 60 °C for 24 h. Ultrathin sections obtained with an ultramicrotome (EM UC7; Leica, Austria) were mounted on a formvar-coated one-hole grid, stained with uranyl acetate and lead citrate, and then examined with a transmission electron microscope (JEM-1400; Jeol, Japan).

### Immunogold transmission electron microscopy

Mouse and human spermatozoa were fixed with 4% (v/v) paraformaldehyde and 0.5% glutaraldehyde in PBS for one hour at room temperature, dehydrated using increasing concentrations of ethanol, embedded in LR White resin (Cat. 02646ab, London Resin Co. Ltd., UK), and subsequently polymerized overnight at 56 °C. Ultrathin (60 nm) sections were mounted on formvar‐covered nickel grids. Thin sections were blocked with 10% fetal bovine serum (Hyclone SH30071.02) for one hour at room temperature and then incubated with an anti-ZO-2 antibody (Cat. 711,400, dilution 1:40, Invitrogen, CA, USA) overnight at 4 °C, followed by a 20 nm gold-conjugated anti-rabbit antibody (Cat. 17,020, dilution 1:50, Ted Pella Inc., CA, USA) for one hour at room temperature. The sections were contrasted with uranyl acetate and lead citrate and observed using a Jeol JEM‐1011 transmission electron microscope (JEOL Ltd., Tokyo, Japan).

### Pull-down assays

Pull-down assays were performed as previously reported (Hernandez-Guzman, et al. 2021). Briefly, HEK293T cells were transfected with amino (398–962 nt), 3PSG (1595–3019 nt), or AP (3029–3923 nt) segments of cZO-2 in the pcDNA4/HisMax vector, as previously reported (Betanzos, et al. 2004). After 24 h, the cells were lysed, and the extracts were subjected to affinity chromatography using Complete His-Tag Purification Columns (Cat. COHISC-RO, Sigma Aldrich, St. Louis, MO, USA), following the manufacturer’s instructions. The purified fractions were analyzed by SDS-PAGE and blotted with antibodies against the 6 × His tag, TPX2, NuMA, and Aurora-A, as indicated in the Western blot section.

### Drugs

Nocodazole (Cat. M-1404, Merck Millipore, Darmstadt, Germany) was prepared as a 10 mM stock in DMSO, and used at a concentration of 0.3 μM. Docetaxel (Cat. 01885, Merck Millipore, Darmstadt, Germany) was prepared as a 10 mM stock in DMSO and used at a concentration of 10 μM. 1,6-hexanediol (Cat. 240,117-50G, Merck Millipore, Darmstadt, Germany) was prepared as a 100% (w/v) stock in DMSO and used at a concentration of 10% (v/v). SAG (Cat. 566,660, Merck Millipore, Darmstadt, Germany) was prepared as a 1.6 mM stock in H_2_O and used at a concentration of 1.6 μM. PI3K inhibitor LY294002 (Cat.PHZ1144, Life Technologies, Carlsbad, CA) was dissolved in DMSO, as a 50 mM stock and used at a concentration of 50 μM. Tubastatin-A (Cat. 15,785, Cayman Chemical, Ann Arbor, MI) was dissolved in DMSO to a concentration of 10 mM and used at a final concentration of 10 μM. Capivasertib (AZD5363) (Cat. S8019, Selleck Chemicals LLC, Houston, TX) was prepared as a 30 mM stock in DMSO and used at a concentration of 0.3 μM Aurora-A Inhibitor I, TCS7010 (Cat.S1451, Selleck Chemicals LLC, Houston, TX) was prepared as a 10 mM stock in DMSO and used at a concentration of 0.1 μm.

## Results

### ZO-2 is present in the centriole, pericentriolar region, and mitotic spindle poles

**Fig. 1:**
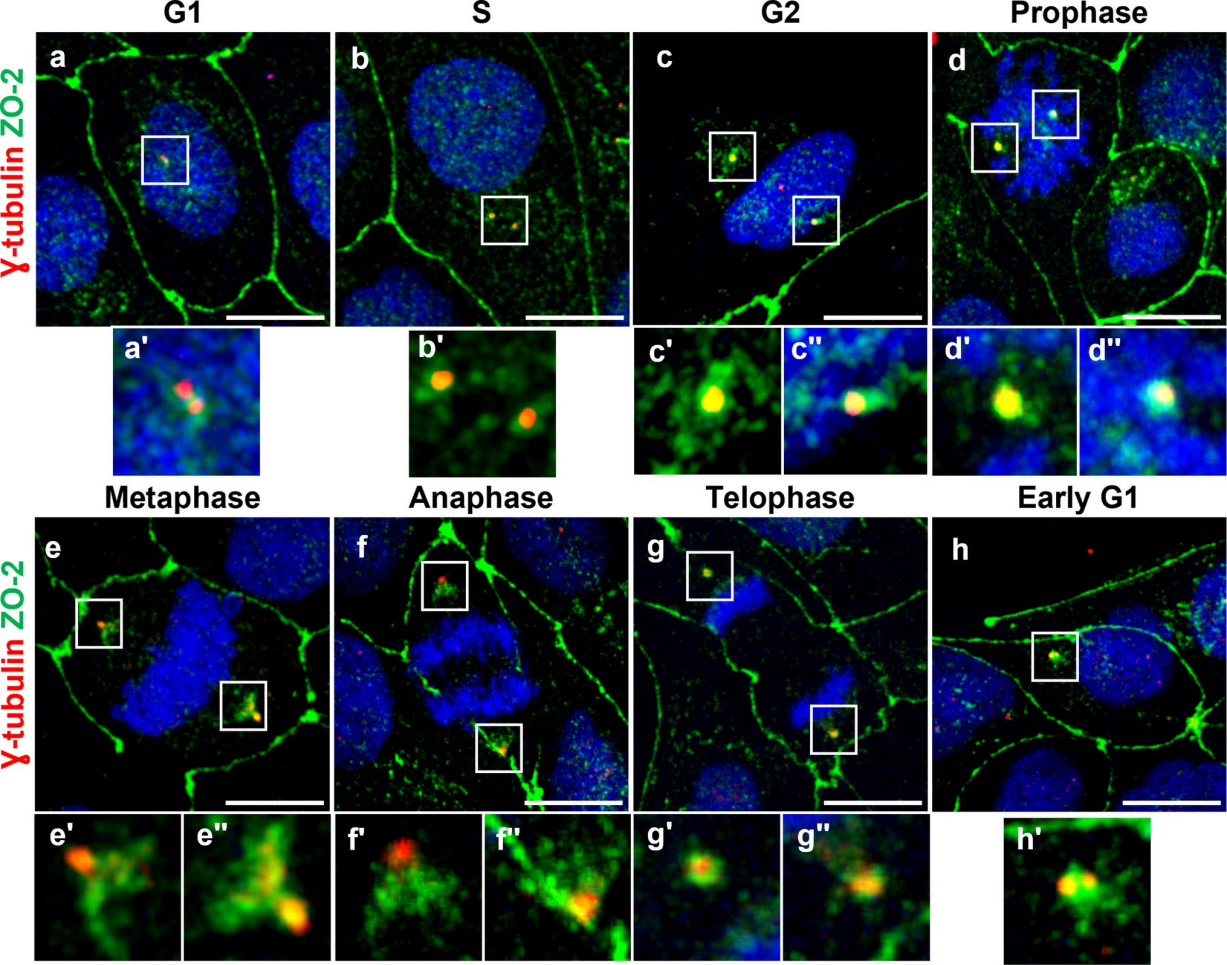
ZO-2 is present in the centrioles and pericentriolar regions at all cell cycle stages. Subconfluent MDCK monolayers were processed for immunofluorescence with antibodies against ZO-2 and γ-tubulin. Nuclei and DNA condensation in chromosomes of cells in mitosis is detected with DAPI. Bar, 10 μm

The relationship between ZO-2 and the centrosome was first studied throughout the cell cycle using immunofluorescence. For this purpose, MDCK cells were co-stained with antibodies against γ-tubulin for centrosome detection and anti-ZO-2 antibodies. Figure 1 shows that ZO-2, regardless of the cell cycle stage, is consistently present in the centrioles and pericentriolar region and, as expected for a TJ protein, is located at the cell borders. In G1, two close spots of γ-tubulin mark the centrioles, while ZO-2 stains both the centrioles and the pericentriolar region (Fig. 1a, a’). During the S phase, the centrioles are farther apart, and ZO-2 staining is maintained at both the centrioles and pericentriolar region (Fig. 1b, b’). At G2 and prophase, the centrioles are positioned at opposite poles and show strong co-localization with ZO-2 (Fig. 1c, c’, c’’ and d, d’, d’’). In metaphase and anaphase, ZO-2 remains at the centrioles but noticeably radiates from the spindle poles in the uppermost section of the presumptive mitotic spindle (Fig. 1e, e’, e’’ and f, f’, f’’). In telophase and early G1, ZO-2 is found in the centrioles and the pericentriolar region (Fig. 1g, g’, g’’, and h, h’).

### ZO-2 concentrates at the DA of the MC

Next, we aimed to analyze through immunofluorescence in MDCK cells whether ZO-2 colocalized with several proteins located in different regions of the centrosome (Supp. Figure 2 a). To achieve this, we first investigated the distribution of markers for the cartwheel, DA, and SDA in the MC. Antibodies against γ-tubulin, a marker of the centrosomal matrix, stained both the MC and DC (Supp. Figure 2 b, c, and d), whereas CEP164, a marker of the DA, stained only the MC (Supp. Figure 2 b). Likewise, staining with antibodies against centriolin, a protein of the SDA, resulted in fluorescent staining confined to the MC (Supp. Figure 2 c). When immunofluorescence was conducted with antibodies against CEP135, a protein of the cartwheel, both centrioles were stained (Supp. Figure 2 d).

**Fig. 2:**
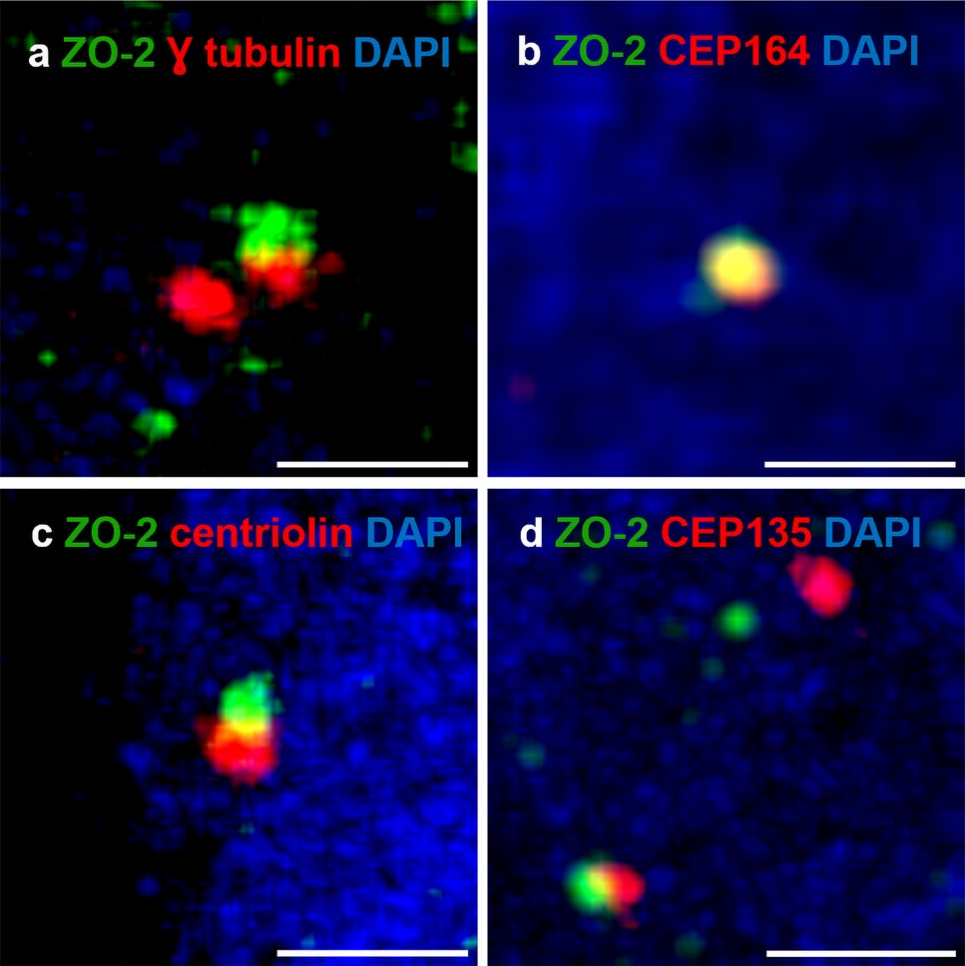
ZO-2 colocalizes with CEP164, a protein present at the DA of the MC. MDCK monolayers were processed for immunofluorescence with antibodies against: **a** ZO-2 and γ-tubulin, (**b**) ZO-2 and CEP164, (**c**) ZO-2 and centriolin, and (**d**) ZO-2 and CEP135. Nuclei were stained with DAPI. Bars, 2 μm

We then analyzed the distribution of ZO-2 in the centrosome. Figure 2a shows that ZO-2 colocalizes with γ-tubulin in only one centriole. To determine the location of ZO-2, we co-stained the cells with antibodies against ZO-2 and the DA protein CEP164, which is found exclusively in the MC. Figure 2b illustrates a clear colocalization of ZO-2 and CEP164, indicating that ZO-2 is situated at the DA of the MC within the centrosome. Next, we investigated whether ZO-2 also colocalizes with centriolin, a protein of the SDA of the MC. Figure 2c shows a partial colocalization of ZO-2 with centriolin, indicated by a narrow yellow mark between the green staining of ZO-2 and the red marking of centriolin. Additionally, a partial colocalization was observed with CEP135 (Fig. 2d), a protein located in the cartwheel. These observations indicate that ZO-2 is present in the MC of the centrosome, concentrating at the DA.

### The lack of ZO-2 alters the cellular content of CEP164, centriolin, and CEP135

Next, we inquired whether the absence of ZO-2 affects the cellular content of centrosomal proteins CEP164, centriolin, and CEP135. Supplemental Fig. 3 A demonstrates, through Western blot analysis of lysates from parental and ZO-2 knock-down (KD) MDCK cells, reduced expression of CEP164 and CEP135, alongside increased centriolin in cells lacking ZO-2. However, these proteins were not significantly recovered after transfecting hZO-2 into ZO-2 KD cells. This may be attributed to the Western blot experiments, which quantified total protein levels in the entire monolayer, whereas only a small percentage of cells within the monolayer were transfected. To improve transfection efficiency, we previously tested electroporation and various transfection reagents. However, none enhanced the transfection efficiency of ZO-2 KD MDCK cells beyond the 20% achieved with the Lipofectamine 2000 reagent used consistently throughout our experiments (Hernandez-Guzman, et al. 2021). We also investigated whether the decrease in CEP164 and the increase in centriolin observed in ZO-2 KD cells were regulated at the transcriptional level. A qRT-PCR analysis revealed no difference in CEP164 mRNA between parental and ZO-2 KD cells, while centriolin mRNA increased in ZO-2 KD cells (Supplemental Fig. 3 B). These results suggest that ZO-2 KD cells have an increased rate of CEP164 degradation and centriolin transcription.

### ZO-2 depletion alters the centrosomal content of CEP164, centriolin, and CEP135

Confocal laser scanning microscopy (CLSM) showed a slight reduction in CEP164 immunofluorescence intensity in the centrosomes of ZO-2 KD cells compared to parental cells, as further demonstrated by quantitative analysis (Supplemental Fig. 3 C). Analysis of CEP164 using SIM super-resolution microscopy revealed no noticeable differences in the immunofluorescence pattern of this protein in the centrosomes between parental and ZO-2 KD cells (Supplemental Fig. 3 D).

Centriolin immunofluorescence in the centrosomes observed by CLSM increased in ZO-2 KD cells compared to parental cells, as confirmed by a quantitative analysis of fluorescence intensity (Supplemental Fig. 3 E). SIM super-resolution microscopy showed no apparent change in the centriolin immunofluorescent pattern (Supplemental Fig. 3 F).

By CLSM immunofluorescence, we observed a decrease in the immunofluorescence intensity of CEP135 in the centrosomes of ZO-2 KD cells compared to parental MDCK cells, as confirmed by quantitative analysis (Supplemental Fig. 3 G).

### ZO-2 depletion has no impact on centriolar structure

Next, we analyzed the appearance of centrosomes using TEM in both parental and ZO-2 KD cells (Supplemental Fig. 4). All longitudinal centrosomal sections examined in parental and ZO-2 KD MDCK cells exhibited classic centrioles (Supplemental Fig. 4 Aa-j). Cross-sectional analysis of the centrosomes indicated a normal distribution of the nine microtubule triplets in parental and ZO-2 KD cells (Supplemental Fig. 4 Ba-j).

These results suggest that the absence of ZO-2 has no apparent impact on the structure of centrioles.

### The lack of ZO-2 concentrates tubulin at the cell borders and reduces microtubule stability and cilia development

The centrosome is the primary microtubule-organizing center, and the SDAs of the centriole are critical for microtubule anchoring, nucleation, and release (Tateishi, et al. 2013) [for reviews, see (Hall and Hehnly 2021; Streubel and Pereira 2023)]. Since ZO-2 KD cells exhibit increased expression of centriolin, we next investigated whether the absence of ZO-2 affects microtubule organization. Microtubules are polymers of α/β-tubulin heterodimers; hence, the expression of α-tubulin in parental and ZO-2 KD cells was analyzed, revealing that a broader band of α-tubulin at the cell borders is observed in ZO-2 KD cells compared to parental cells (Supplemental Fig. 5 A). This was further confirmed by the intensity profiles obtained from a line scan across the cell borders, which showed that ZO-2 KD cells display a broader peak of immunofluorescence at the cell borders compared to parental cells (Supplemental Fig. 5 B). However, quantification of fluorescence intensity across the entire field showed no significant difference between parental and ZO-2 KD cells (Supplemental Fig. 5 C). These results indicate that the absence of ZO-2 induces a rearrangement of microtubules at the cell borders.

**Fig. 3:**
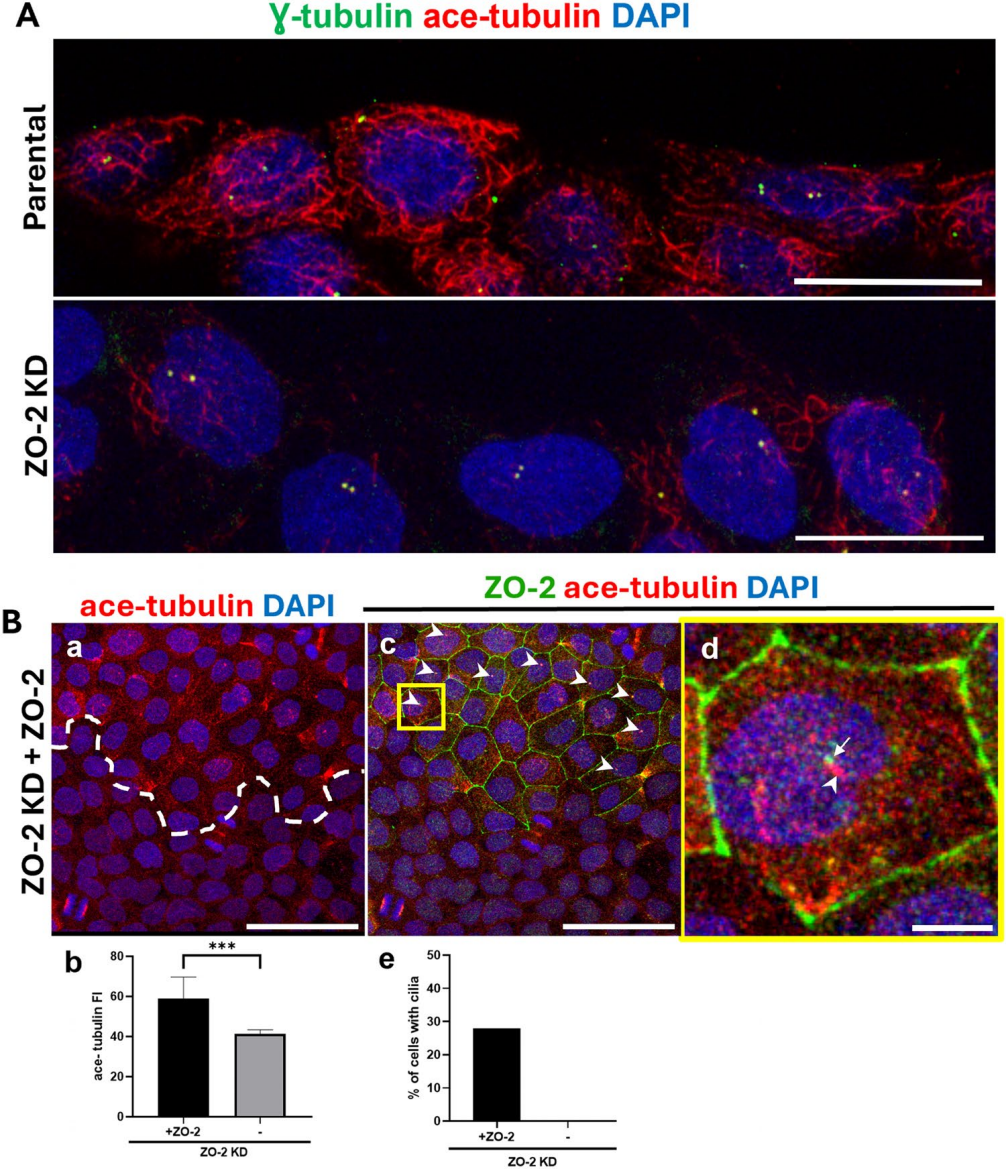
The lack of ZO-2 inhibited microtubule stabilization, (**A**) The lack of ZO-2 triggers a reduction of stable microtubules stained with acetylated tubulin in wound-edge migrating cells. Parental and ZO-2 KD cells were fixed 6 h after making a scratch with a pipette tip. Then, the monolayers were stained with DAPI and antibodies against γ-tubulin and acetylated tubulin to detect the centriole and the stable microtubules, respectively. Representative images of two independent experiments. Bars, 20 μm. **B** The re-expression of ZO-2 in ZO-2 KD cells induces a higher abundance of acetylated microtubules than ZO-2 KD cells. ZO-2 KD cells that re-express ZO-2 were processed for immunofluorescence with antibodies against ZO-2 and acetylated tubulin (ace-tubulin). Nuclei were stained with DAPI. a The white dashed line indicates the border between ZO-2 re-expressing and non-expressing cells in a ZO-2 KD cell culture. Bar, 50 μm. b Quantitation of total immunofluorescence intensity (FI) of acetylated tubulin in a culture of ZO-2 KD cells in regions where ZO-2 is re-expressed or not. Ten optical fields were analyzed. Statistical analysis was done using the Student’s* t* test with Welch’s correction. ***p < 0.001. **c** ZO-2 staining at the cell borders identifies the regions where ZO-2 is re-expressed in a ZO-2 KD monolayer. Arrowheads indicate developing cilia in ZO-2 KD cells re-expressing ZO-2. The yellow frame corresponds to the area amplified in d. Bar, 50 μm. **d** A developing cilia is observed in ZO-2 KD cells re-expressing ZO-2. Arrowhead, acetylated tubulin in the axoneme of developing cilia; arrow, ZO-2 at the basal body of cilia. Bar, 5 μm. **e** Quantitation of the percentage of cells with cilia in ZO-2 KD cells that re-express ZO-2 or not. 100 cells from each group were analyzed

Tubulin acetylation produces stable microtubules that are more resistant to depolymerization when exposed to cold temperatures and when treated with depolymerizing drugs. Additionally, acetylation helps prevent microtubules from mechanical aging [for review, see (Carmona, et al. 2023)]. Wound-edge migrating cells feature stable acetylated microtubule arrays concentrated around the centrosome (Hung, et al. 2016). Therefore, testing was conducted to determine whether the absence of ZO-2 altered this pattern. Figure 3A shows that the abundance of acetylated microtubule arrays decreases in wound-edge migrating cells of ZO-2 KD cells compared to parental monolayers. To further assess whether ZO-2 is necessary for microtubule stabilization, the expression of acetylated microtubules in ZO-2 KD cells that re-express ZO-2 was analyzed. Figure 3B illustrates that the ZO-2 KD cells in the monolayer that re-express ZO-2 show a greater abundance of acetylated microtubules than ZO-2 KD cells. Additionally, we observed that 28% of ZO-2 KD cells that re-express ZO-2 develop cilia, whereas ZO-2 KD cells do not (Fig. 3Bc, d, e). This reversion/rescue of the phenotype experiment indicates that ZO-2 is required for microtubule stabilization and cilia development.

### The lack of ZO-2 inhibits the development of astral and mitotic spindle microtubules stained with EB1

Previous studies have indicated that ZO-2 depletion causes mitotic spindle disorientation, contributing to the formation of multiple lumens per cyst in 3D cultures (Raya-Sandino, et al. 2017). Therefore, we next examined whether the absence of ZO-2 affected the development of astral microtubules in mitotic cells.

**Fig. 4:**
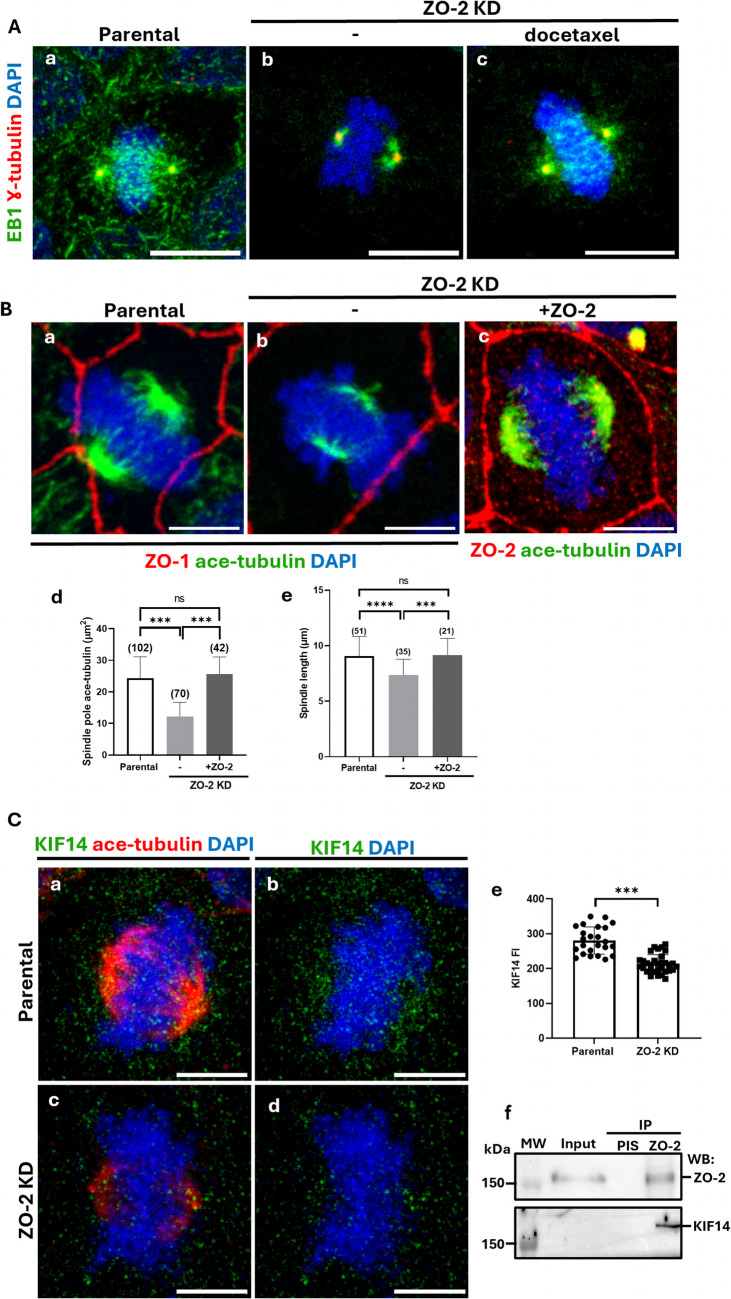

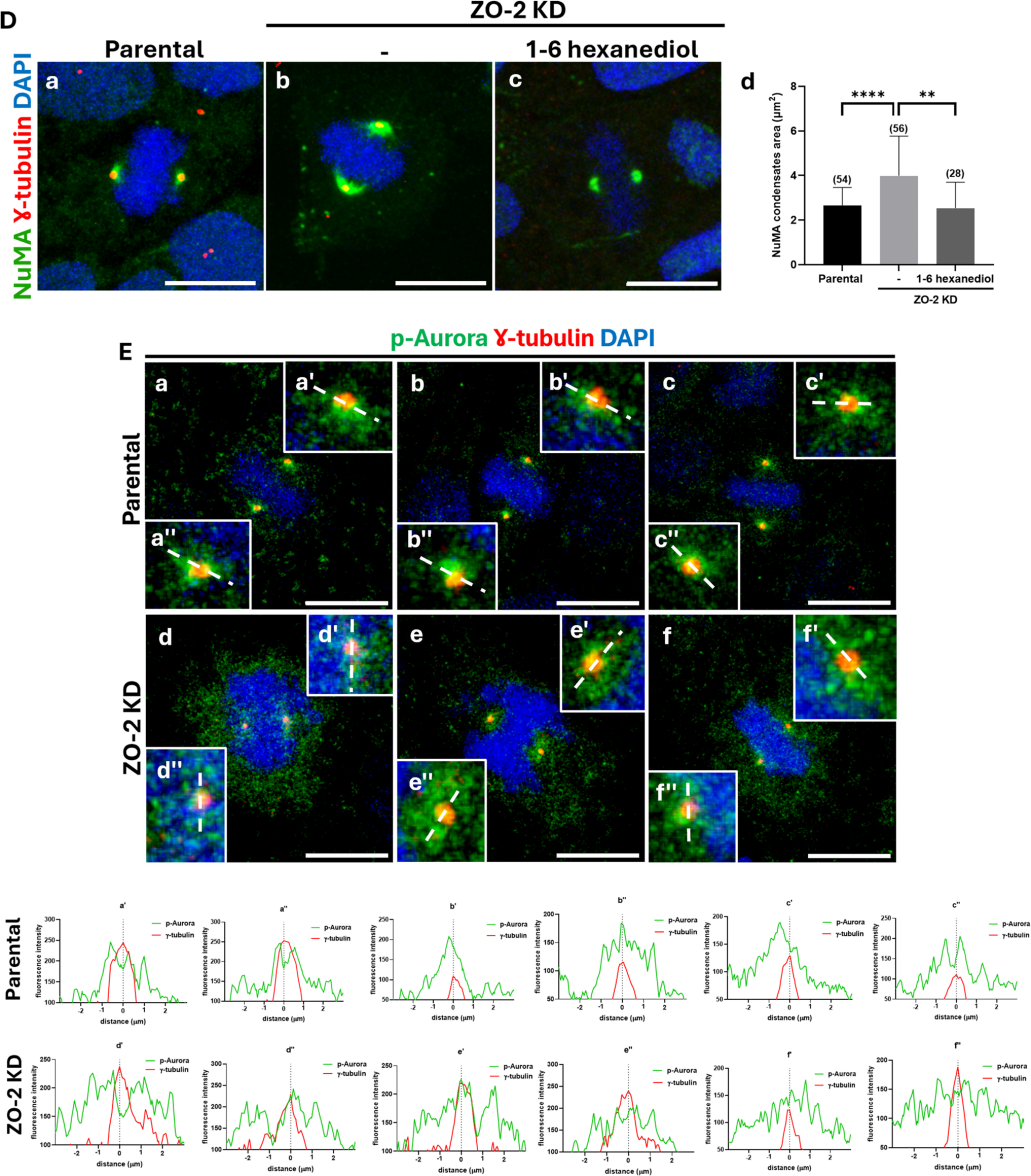
The lack of ZO-2 blocked the development of astral and mitotic spindle microtubules, reduced the spindle length, and altered the accumulation at the spindle poles of acetylated tubulin, NuMA, p-Aurora, KIF14, and TPX2. **A** The lack of ZO-2 inhibits the localization of EB1 in astral microtubules and the mitotic spindle. Subconfluent parental and ZO-2 KD cells treated or not with docetaxel were processed for immunofluorescence with DAPI to stain their DNA and with antibodies against γ-tubulin and EB1, a plus-end microtubule marker. Representative images of two independent experiments. Bars, 10 μm. **B** The concentration of acetylated tubulin at the spindle poles and the mitotic spindle length are reduced in ZO-2 KD MDCK cells. Sparse cultures of parental and ZO-2 KD MDCK cells that re-express or not ZO-2 were fixed and processed for immunofluorescence with DAPI to stain their DNA, with antibodies against ZO-1 or ZO-2 to stain the cell border, and with antibodies against acetylated tubulin to detect the mitotic spindle poles. (a,b and c) Representative images of two independent experiments. Bars, 5 μm. d) Quantitation of the area of acetylated tubulin present at the spindle poles. Statistical analysis was done using the Mann–Whitney test. Numbers in parentheses indicate the number of spindle poles analyzed per condition. ***p < 0.001. e) Quantification of the spindle length. Statistical analysis was done using the Mann–Whitney test. Numbers in parentheses indicate the number of spindle poles analyzed per condition. ***p < 0.001, ****p < 0.0001. **C** ZO-2 depletion reduces the expression of kinesin KIF14 at spindle poles. Subconfluent parental and ZO-2 KD cells were processed for immunofluorescence using DAPI to stain their DNA and rabbit antibodies against acetylated tubulin, and a mouse monoclonal anti-KIF14 antibody. A secondary antibody against rabbit IgG, coupled to Alexa594, was employed in conjunction with a mouse IgGκ light chain binding protein, coupled to FITC. **a-d** Representative images of two independent experiments. **e** Quantification of KIF14 fluorescent intensity. Statistical analysis was done using Student’s *t* test. ***p < 0.001. Bars, 5 μm. **f** KIF14 is detected by Western blot in a ZO-2 immunoprecipitate derived from suconfluent parental cells. **D** The absence of ZO-2 triggers the accumulation of NuMA condensates in the spindle poles. Subconfluent parental (a) and ZO-2 KD (b, c) MDCK cells were incubated or not for 60 s with 10% 1,6-hexanediol. They were then fixed and processed for immunofluorescence with DAPI to stain their DNA, along with antibodies against γ-tubulin and NuMA, a minus-end microtubule marker. Representative images of two independent experiments. Bars, 10 μm. Statistical analysis was done with the Mann–Whitney test (d). Numbers in parenthesis indicate the number of spindle poles analyzed in parental and ZO-2 KD cells **p < 0.01, ****p < 0.0001. **E** In ZO-2 KD cells, active Aurora exhibits a more diffuse distribution around the spindle pole than in parental cells. Sparse parental and ZO-2 KD MDCK cells were fixed and processed for immunofluorescence with DAPI to stain their DNA, with antibodies against γ-tubulin to stain the centriole in the mitotic spindle poles, and with antibodies against phosphorylated Aurora (p-Aurora) to detect the active form of the kinase. Upper panel, representative images of three independent experiments. Bars, 10 μm. In the lower panel, linescan analysis with fluorescence intensities of the two channels is performed as a function of distance from the midline γ-tubulin labeling

Figure 4Aa reveals that in parental proliferating cells, EB1, a protein that associates with the plus-end of active polymerizing microtubules, localizes around the centrosomes stained with γ-tubulin, in the astral microtubules radiating from the spindle poles, and along the mitotic spindle. In contrast, in ZO-2 KD cells, EB1 surrounded the centrosomes at the spindle poles but was absent in the astral microtubules or along the mitotic spindle (Fig. 4Ab). The stabilization of microtubules with 10 µM docetaxel partially rescues EB1 localization in the astral microtubules and the mitotic spindle of ZO-2 KD cells (Fig. 4Ac), suggesting that ZO-2 plays a crucial role in maintaining microtubule stability, which is essential for the proper development of astral and mitotic spindle microtubules.

### Mitotic ZO-2 KD cells exhibit abnormal chromosome congression, show reduced spindle length, and display less acetylated tubulin and a lower accumulation of KIF14 at the spindle poles

To further investigate whether the absence of ZO-2 affected the stability and appearance of the mitotic spindle, proliferating monolayers of parental and ZO-2 KD cells were stained with antibodies against acetylated tubulin. In ZO-2 KD cells, the amount of acetylated/stable tubulin at the spindle pole decreased compared to parental cells (Fig. 4Ba,b). However, when ZO-2 KD cells re-express ZO-2, the level of acetylated tubulin at the spindle poles is restored (Fig. 4Bc). This observation was confirmed by quantifying the area occupied by acetylated tubulin at the spindle poles (Fig. 4Bd). These observations suggest that ZO-2 plays a crucial role in maintaining microtubule stability within the mitotic spindle.

As expected, the reduced microtubule stability of the mitotic spindle in ZO-2 KD cells was associated with a shorter spindle length compared to that observed in parental cells (Fig. 4Ba,b). Following ZO-2 re-expression, ZO-2 KD cells displayed a mitotic spindle length akin to that of parental cells (Fig. 4Bc). The quantification of spindle length supported these findings (Fig. 4Be). Additionally, we noted abnormal chromosome congression in ZO-2 KD cells during mitosis (Fig. 4Bb).

Kinesin-14 is a motor protein that moves along microtubules from their plus to minus ends. It has microtubule-organizing functions, including the crosslinking of parallel microtubules, the sliding of antiparallel microtubules, and the clustering of minus ends, which leads to the organization of spindle poles (Simeonov, et al. 2009; Walczak, et al. 1997). Silencing Kinesin-14a (KIF14) generates shorter, thinner spindles (Cai, et al. 2009) and causes abnormal chromosomal congression (Zhu, et al. 2005). Since these changes resemble those observed in ZO-2 KD cells, we next analyzed the expression of KIF14 in the mitotic parental and ZO-2 KD cells. Figure 4C reveals a diminished concentration of KIF14 at the mitotic spindle poles of ZO-2 KD cells compared to parental cells. In ZO-2 KD cells, a very strong reduction of acetylated tubulin in the spindle poles is also observed.

Additionally, we observed that in MDCK cells, KIF14 co-immunoprecipitated with ZO-2 (Fig. 4Cf), consistent with previous proximity-dependent biotin identification assays, as well as affinity and mass spectrometry experiments that revealed the interaction between ZO-2 and KIF14 (Capalbo, et al. 2019, Cho, et al. 2022, Couzens, et al. 2013, Go, et al. 2021). Altogether, these results suggest that KIF14 interaction with ZO-2 favors its accumulation at the mitotic spindle poles.

### The spindle poles in ZO-2 KD cells display an increase in NuMA condensates and a reduced accumulation of active Aurora-A

The length of the mitotic spindle is regulated by the poleward flux of spindle microtubules, where tubulin subunits are continually added at the plus-end and flow to the minus-end, where they are removed through depolymerization mediated by kinesin-13 family members, including Kif2A. NuMA (Nuclear Mitotic Apparatus) is a microtubule-associated protein that accumulates at mitotic spindle poles and regulates spindle assembly and dynamics via liquid–liquid phase separation (Sun, et al. 2021). NuMA condensates at the spindle poles concentrate Kif2A, which depolymerizes spindle microtubules and promotes poleward flux. Consequently, cells that overexpress NuMA exhibit a reduction in spindle length (Sun, et al. 2021). Therefore, an analysis was performed to determine whether the absence of ZO-2 affected NuMA expression at mitotic spindle poles. Figure 4D illustrates that in ZO-2 KD cells, the concentration of NuMA at the spindle poles increases compared to that in parental cells.

Since NuMA droplets concentrate Kif2A, which promotes the poleward spindle microtubule flux (Sun, et al. 2021), a test was conducted to determine if the observed NuMA concentrates present at the spindle poles of ZO-2 KD cells are indeed condensates formed by liquid–liquid phase separation. For this purpose, NuMA's sensitivity to 1,6-hexanediol was tested, as this compound destroys and inhibits the formation of liquid-like droplets through the disruption of hydrophobic interactions (Sun, et al. 2021). Figure 4Dc shows that NuMA presence at spindle poles significantly diminished when ZO-2 KD cells were incubated for 60 s with 10% 1,6-hexanediol, indicating that NuMA accumulation at the poles in ZO-2 KD cells is generated by liquid–liquid phase separation. Quantification of NuMA condensates confirms that the increase in condensate area observed in ZO-2 KD cells is abolished after treatment with 1–6 hexanediol (Fig. 4Dd).

A reduced spindle length was observed when NuMA present at the spindle poles, was not phosphorylated at S1969 by the kinase Aurora-A (Sun, et al. 2021). Consequently, the accumulation of active Aurora (p-Aurora) in the spindle poles of mitotic cells was examined. In parental cells, p-Aurora is concentrated around the mitotic spindle poles. In contrast, in ZO-2 KD cells, the active kinase showed a more dispersed and less concentrated distribution at the spindle poles (Fig. 4E upper panel), as confirmed by the intensity profiles obtained from a linescan across the spindle poles (Fig. 4E, lower panel). Western blot analysis revealed no change in the total amount of p-Aurora between parental and ZO-2 KD MDCK cells (Suppl. Figure 6), indicating that the absence of ZO-2 impacts active Aurora recruitment to mitotic spindle poles but does not alter the total cellular content.

In summary, the observations indicate that in mitotic cells, the absence of ZO-2 at the spindle poles concentrates NuMA and reduces the accumulation of active Aurora, suggesting an increase in the poleward spindle microtubule flux that, together with the decreased accumulation of KIF14 and the instability of microtubules, decreases the length of the mitotic spindle.

### ZO-2 is needed to concentrate TPX2 at mitotic spindle poles

During mitosis, a Ran-GTP gradient is present near the chromosomes [for review, see (Kufer, et al. 2003)]. Ran-GTP releases TPX2, a spindle assembly factor, from importin α, allowing TPX2 to associate with Aurora-A. This interaction enables Aurora-A to orient its autophosphorylated threonine residue inward and to protect it from dephosphorylation by phosphatase PP1 (Bayliss, et al. 2003). Hence, TPX2 binding locks Aurora-A in an active conformation. TPX2 targets Aurora-A to mitotic spindle poles, but not to centrosomes (Kufer, et al. 2002), and favors the interaction between active Aurora-A and NuMA (Polverino, et al. 2021). Aurora-A, TPX2, and NuMA form a protein complex at the mitotic spindle poles, where the phosphorylation of NuMA by Aurora-A leads to its movement from the spindle pole to the cell cortex during metaphase (Polverino, et al. 2021).

**Fig. 5:**
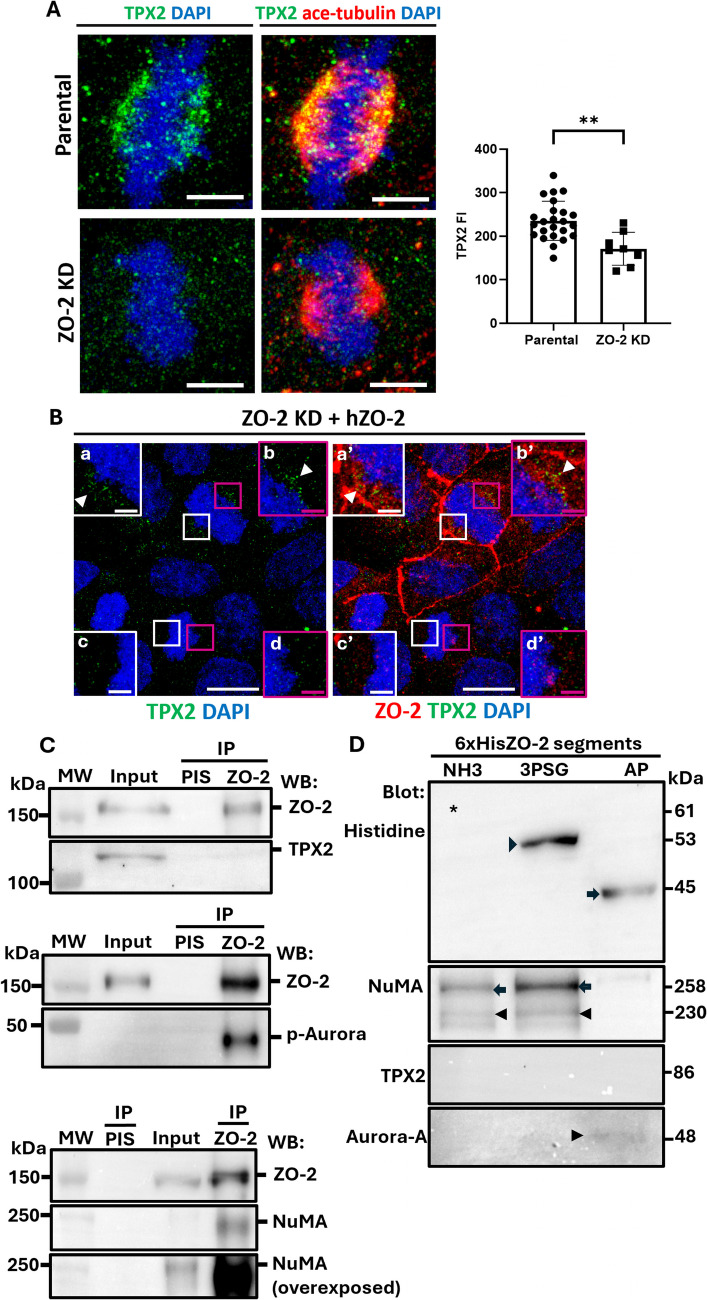
ZO-2 associates with Aurora-A and NuMA, and its depletion reduces TPX2 concentration at mitotic spindle poles. **A** In ZO-2 depleted cells, TPX2 accumulation at spindle poles diminishes. Sparse parental and ZO-2 KD MDCK cells were fixed and processed for immunofluorescence using DAPI to stain the chromosomes, antibodies against acetylated tubulin for the mitotic spindle poles, and antibodies against TPX2. The left panel shows representative images from three independent experiments. Bars, 5 μm. The right panel presents a quantitative analysis of the immunofluorescence intensity of TPX2 at the spindle poles, with statistical analysis conducted using the Mann–Whitney test. **p < 0.01. **B** Re-expressing ZO-2 in ZO-2 KD cells restores TPX2 expression at the mitotic spindle poles. ZO-2 KD cells re-expressing ZO-2 were processed for immunofluorescence using antibodies against ZO-2 and TPX2. Nuclei were stained with DAPI. Bars 10 μm, inserts bars 2 μm. **C** NuMA and p-Aurora, but not TPX2, co-immunoprecipitated with ZO-2 from a lysate derived from proliferating parental cells. PIS, pre-immune serum. **D** Aurora-A and NuMA bind to different segments of ZO-2. 6xHis-tagged amino (NH3), 3PSG, and AP segments of cZO-2 were purified with Ni affinity columns from extracts of HEK293T cells, run in SDS-PAGE, and blotted with antibodies against the histidine tag, TPX2, NuMA, and Aurora-A. Upper panel, * band of 61 kDa of amino ZO-2 segment; full arrowhead, band of 53 kDa of 3PSG ZO-2 segment; full arrow, band of 45 kDa of AP ZO-2 segment. Lower three panels: Aurora-A gives a faint positive signal in the pull-down of the AP segment of cZO-2. NuMA is present in the pull-downs of the amino and 3PSG segments of cZO-2, albeit with a stronger signal for the 3PSG segment. Full arrowhead, band of 230 kDa of NuMA; full arrow, band of 258 kDa corresponding to a heavily phosphorylated NuMA. In contrast, TPX2 was not detected in any of the pull-downs of cZO-2 segments

A proximity-dependent biotin identification assay previously demonstrated the interaction of ZO-2 with TPX2 (Huttlin, et al. 2021). Therefore, we next analyzed whether the absence of ZO-2 altered the concentration of TPX2 at the mitotic spindle poles. Figure 5A reveals decreased expression of TPX2 at the spindle poles of ZO-2 KD cells compared to parental cells. Likewise, in ZO-2 KD cells that re-express ZO-2, TPX2 staining at the spindle poles increases (Fig. 5B a,a’,b,b’) compared to cells where ZO-2 is not expressed (Fig. 5B c,c’,d,d’). These results suggest that ZO-2 at the spindle pole may act as a platform that facilitates the concentration of TPX2.

### At the mitotic spindle pole, ZO-2 works as a scaffold for Aurora-A and NuMA

Since ZO-2 KD cells exhibit a decreased amount of TPX2 and a diminished concentration of Aurora-A at the spindle poles alongside an increased abundance of NuMA condensates, we hypothesized that ZO-2 may serve as a scaffold at the spindle pole, supporting the TPX2/Aurora-A/NuMA complex. To investigate this, we immunoprecipitated ZO-2 from parental cells during proliferation and detected the co-immunoprecipitation of Aurora-A and NuMA, but not TPX2, by Western blot (Fig. 5C).

To further identify which segments of ZO-2 associate with TPX2, Aurora-A, and NuMA, we conducted a pull-down assay using the human kidney cell line HEK293T. For this purpose, the cells were transfected with constructs of cZO-2: the amino (coding for PDZ domains 1, 2, and 3), 3PSG (coding for PDZ-3, SH3, and GuK domains), or AP (coding for the acidic and proline-rich regions), all introduced in the pcDNA4/HisMax vector. The synthesized peptides were purified from extracts of HEK293T cells using Ni affinity columns, subjected to SDS-PAGE, and blotted with antibodies against the histidine tag. The upper panel of Fig. 5D shows bands at 62, 53, and 45 kDa, corresponding to the amino, 3PSG, and AP segments of cZO-2. Blotting with antibodies against TPX2, Aurora-A, and NuMA revealed that Aurora-A and NuMA, but not TPX2, are present in the pull-down of ZO-2 (Fig. 5D, lower three panels). Aurora-A was associated with the AP segment of ZO-2, while NuMA was detected in the pull-downs of the amino and 3PSG segments of ZO-2. Since the latter segments share the PDZ-3 domain of ZO-2 in common, this result suggests that NuMA binds to the PDZ-3 domain of ZO-2. However, since the signal of NuMA in the 3PSG segment is stronger than that in the amino segment, the presence of the contiguous supradomain of SH3-GuK might provide stability for this interaction.

These results indicate that at the spindle pole, ZO-2 acts as a scaffold for the TPX2/Aurora-A/NuMA complex by associating with both Aurora-A and NuMA. This interaction may promote the phosphorylation of NuMA by Aurora-A and its subsequent displacement to the cell cortex, which could explain why NuMA accumulates in the spindle poles of ZO-2-depleted cells, while p-Aurora-A is dispersed and TPX2 expression is significantly reduced.

### ZO-2 is required for the formation of the primary cilium

**Fig. 6:**
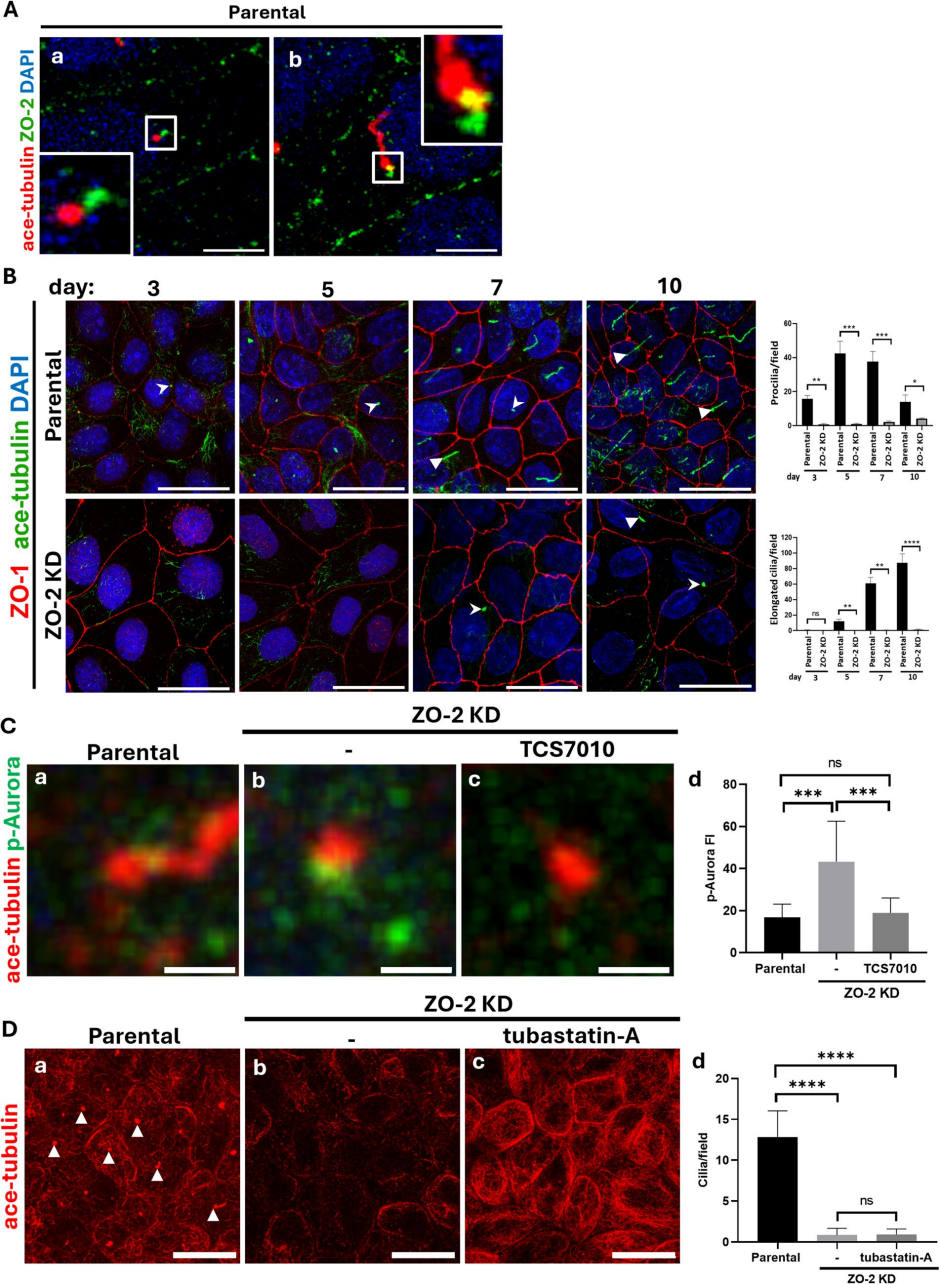
ZO-2 is present at the ciliary base, and its depletion induces p-Aurora accumulation. **A** ZO-2 is present at the basal body of primary cilia. Parental MDCK cells were incubated with antibodies against ZO-2 and acetylated tubulin that stains the axoneme of procilia (a) and elongated primary cilium (b). Nuclei were stained with DAPI. Bar, 5 μm. **B** The lack of ZO-2 blocks the development of primary cilia. Parental and ZO-2 KD MDCK monolayers were fixed and processed for immunofluorescence on different days after plating, with antibodies against acetylated tubulin and ZO-1 for detecting cilia and cell borders. Nuclei were stained with DAPI. Bars, 20 μm. Left panel, representative images. The right panel is a quantitative analysis from 5 optical fields of each condition, derived from two independent experiments. Statistical analysis was done using the student's *t*-test. ns non-significant, *p < 0.05, **p < 0.01, ***p < 0.001, ****p < 0.0001.** C** p-Aurora accumulates at the base of procilia in ZO-2 KD cells. Parental and ZO-2 KD MDCK cells were plated to confluence and incubated in CDMEM for 24 h. The monolayers were then transferred to DMEM without serum to stimulate cilium development, with or without 0.1 μM TCS7010, an Aurora-A inhibitor. After 48 h, the monolayers were fixed and processed for immunofluorescence with antibodies against p-Aurora and acetylated tubulin. Representative immunofluorescence images of p-Aurora at the base of cilia and procilia in parental (a) and ZO-2 KD MDCK cells (b) Bars, 1 μm. c Treatment of ZO-2 KD cells with TCS7010 inhibits the expression of p-Aurora at the ciliary base but does not restore primary cilia expression. Bar, 1 μm. d Quantification of p-Aurora immunofluorescence intensity at the ciliary basal body. At least 12 cilia or procilia were analyzed per experimental condition. Statistical analysis was done using the Mann–Whitney test, ns non significant, ***p <0.001. **D** The inhibition of HDAC6 does not reverse cilia depletion induced by the lack of ZO-2. Parental and ZO-2 KD MDCK cells were plated to confluence and incubated in CDMEM for 24 h. The monolayers were then transferred to DMEM without serum to stimulate cilium development, and a group of ZO-2 KD monolayers was also treated with 10 µM tubastatin-A, an HDAC6 inhibitor. After 48 h, the monolayers were fixed and processed for immunofluorescence with antibodies against acetylated tubulin. a-c Representative immunofluorescence images of cilia and procilia in parental and ZO-2 KD MDCK cells. Bars, 20 μm. Panel d, quantification of cilia/field. At least 12 optical fields were analyzed per experimental condition. Statistical analysis was done using the Mann–Whitney test, ns non significant, ****p <0.0001

Primary cilia are non-motile, antenna-like structures that project from the apical surface of epithelial cells. The mature centriole serves as the basal body or platform for forming primary cilia (Graser, et al. 2007) in non-proliferating cells. Specifically, the DAs of the MC are crucial for initiating primary cilia biogenesis [(Tateishi, et al. 2013), for review, see (Streubel and Pereira 2023)]. ZO-2 in parental MDCK cells is located at the base of procilia and elongated cilia, identified through acetylated tubulin staining (Fig. 6A). Consequently, we examined the effect of ZO-2 depletion on cilia development over time. For this study, parental and ZO-2 KD MDCK cells were fixed on different days after plating at confluent density and processed for immunofluorescence to detect procilia, which are short (< 3 μm in length) acetylated tubulin-positive structures, and elongated primary cilia, which are typically > 3 μm in length (Sfakianos, et al. 2007). Figure 6B shows a significant delay in procilia development in ZO-2 KD MDCK cells compared to parental cells, along with a complete absence of fully elongated cilia in the cells lacking ZO-2.

### ZO-2 depletion induces Aurora-A stabilization at the ciliary basal body

To understand why ZO-2 depletion hinders primary cilium development, we examined the basal bodies of procilia using TEM. No morphological differences were observed between the basal bodies in parental and ZO-2 KD cells (Suppl. Figure 7), indicating that ZO-2 depletion does not prevent the correct docking of the MC at the plasma membrane. Therefore, the absence of ZO-2 does not block cilia development by affecting the centriole structure.

Consequently, we investigated whether the lack of cilia development observed after ZO-2 depletion was due to altered axoneme elongation resulting from microtubule instability. We found that cilia formation could not be induced in ZO-2 KD cells after 24 h of serum starvation, even with microtubules stabilized against depolymerization using 10 µM docetaxel (Suppl. Figure 8).

Instead, we observed that ZO-2 depletion led to the accumulation of p-Aurora-A at the basal body, a condition not observed in parental cells (Fig. 6Ca, b, d). To further test the role of p-Aurora-A on the inhibition of cilia development in ZO-2 depleted cells, ZO-2 KD cells were incubated for 48 h in serum-depleted media with 0.1 μM TCS7010, an inhibitor of Aurora-A. Figures 6Cc and d show that although this treatment inhibited the expression of p-Aurora-A at the basal body, it did not restore the expression of primary cilia in ZO-2 KD cells.

Aurora-A at the basal body phosphorylates and activates the histone deacetylase HDAC6 (Pugacheva, et al. 2007), which subsequently deacetylates tubulin (Hubbert, et al. 2002), obstructing primary cilium formation. However, treating ZO-2 KD cells with 10 µM tubastatin-A, an inhibitor of HDAC6 (Gradilone, et al. 2013), for 48 h did not reverse the cilia depletion observed in ZO-2 KD cells maintained in serum-starved media (Fig. 6D). An increase in acetylated tubulin confirmed the effect of tubastatin-A on tubulin observed in ZO-2 KD-treated monolayers.

Since inhibiting p-Aurora-A or HDAC6 was insufficient to restore cilia formation in ZO-2 KD cells, we investigated whether the expression of other ciliary proteins was also altered in ZO-2-depleted cells.

### ZO-2 depletion diminishes the expression of CEP164, IFT-B protein IFT57, and kinesin KIF14 in procilia

**Fig. 7:**
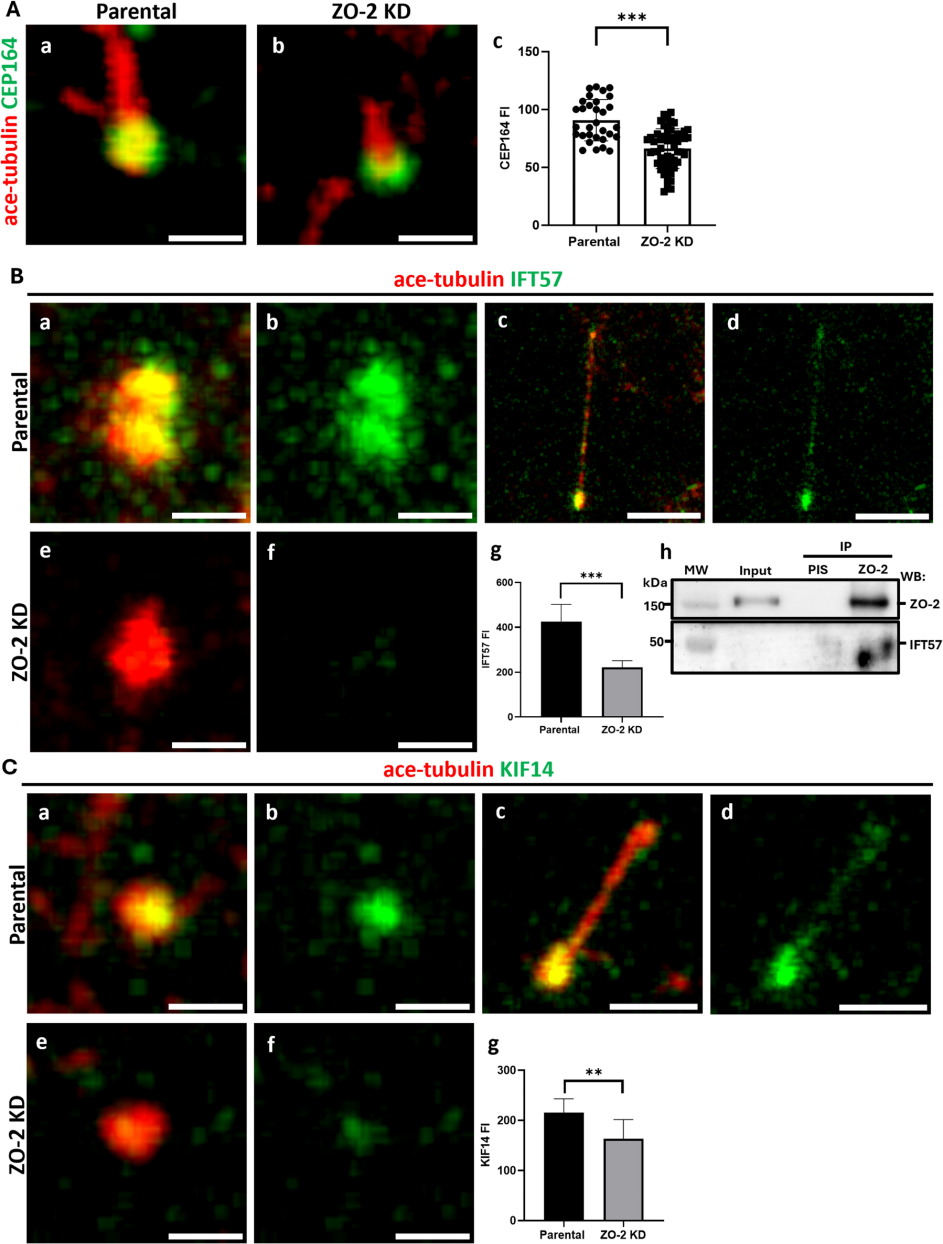
The amount of CEP64, IFT-B protein IFT57, and kinesin KIF14 diminishes at the base of the procilia in ZO-2 KD cells. Parental and ZO-2 KD MDCK cells were plated to confluence and incubated in CDMEM for 24 h. The monolayers were then transferred to DMEM without serum to stimulate cilium development. After 48 h, the monolayers were fixed and processed for immunofluorescence using antibodies against acetylated tubulin and CEP164 (**A**), IFT57 (**B**), or KIF14 (**C**). **A** The concentration of CEP164 at the ciliary base diminishes in ZO-2-depleted cells in comparison to parental cells. Panels a and b, representative immunofluorescence images of CEP164 present in the base of cilia and procilia in parental and ZO-2 KD MDCK cells. Bars 1 μm. Panel c, quantification of CEP164 immunofluorescent intensity in the ciliary base. The statistical analysis was done using the Mann–Whitney test. ***p < 0.001. **B** In parental cells, IFT57 concentrates at the ciliary base and the ciliary tip. The accumulation of IFT57 at the ciliary base diminishes in ZO-2KD cells. Panels a,b,e, and f, representative immunofluorescence images of IFT57 present in the base of procilia in parental (a,b) and ZO-2 KD (e,f) MDCK cells. Bars, 1 μm. Panels c and d, representative immunofluorescence images showing the concentration of IFT57 in the base and tip of cilia present in parental MDCK cells. Bars, 5 μm. Panel g, quantification of IFT57 immunofluorescent intensity in the ciliary base of parental and ZO-2 KD cells. The statistical analysis was done using the Mann–Whitney test. ***p < 0.001. h) IFT57 is detected by Western blot in a ZO-2 immunoprecipitate derived from confluent parental MDCK cells. **C** In parental cells, KIF14 is present at the ciliary base and along the ciliary axoneme. The accumulation of KIF14 at the ciliary base diminishes in ZO-2KD cells. Panels a,b,e, and f, representative immunofluorescence images of KIF14 present in the base of procilia in parental (a,b) and ZO-2 KD (e,f) MDCK cells. Bars, one μμm. Panels c and d, representative immunofluorescence images of KIF14 present in the ciliary base and along the axoneme of elongated cilia in parental MDCK cells. Bars, 2 μm. Panel g, quantification of KIF14 immunofluorescent intensity in the ciliary base of parental and ZO-2 KD cells. The statistical analysis was done using the Mann–Whitney test. **p < 0.01

CEP164, which colocalizes with ZO-2 in the MC (Fig. 2b), is essential for primary cilia formation (Graser, et al. 2007, Pejskova, et al. 2020) as it facilitates vesicular docking to the MC, a critical step in early ciliogenesis (Schmidt, et al. 2012). Therefore, we investigated whether the expression of CEP164 in the primary cilia of ZO-2 KD cells was altered. Figure 7A shows that the level of CEP164 expression at the base of the procilia of ZO-2 KD cells is lower than that observed in the cilia of parental cells.

Next, we analyzed whether the decreased axoneme elongation in the cilia of ZO-2 KD cells was also due to disrupted intraflagellar transport (IFT) machinery. This process involves the anterograde movement of structural components of cilia, such as tubulin, from the ciliary base to the tip of the cilium and the retrograde return of membrane proteins and signaling factors to the ciliary base. IFT trains consist of IFT-A and IFT-B complexes, which contain 6 and 16 different proteins, respectively [for reviews, see (Nakayama and Katoh 2018; Prevo, et al. 2017; Taschner and Lorentzen 2016)]. The IFT-B complex is responsible for anterograde protein trafficking mediated by kinesin-2 motor proteins, while the IFT-A complex facilitates retrograde trafficking through dynein.

Since a deficiency in IFT-B proteins often results in very short cilia or a complete absence of flagella development (Brazelton, et al. 2001; Hou, et al. 2007; Pazour, et al. 2000), we next analyzed the expression of the IFT-B protein IFT57 in primary cilia. This protein, as determined by a proximity-dependent biotin identification assay, associates with ZO-2 (Go, et al. 2021). IFT57 concentrates at the base and tip of primary cilia in parental cells (Fig. 7B a-d). In contrast, the accumulation of IFT57 at the ciliary base decreases in ZO-2 KD cells compared to parental cells (Fig. 7Be,f). The quantification of IFT57 immunofluorescence at the ciliary base of procilia confirms reduced expression in ZO-2 KD cells compared to parental cells (Fig. 7Bg). In addition, we found that IFT57 is present in a ZO-2 immunoprecipitate derived from confluent parental cells (Fig. 7Bh), thus confirming the interaction of ZO-2 with IFT57.

The depletion of KIF14 increases the level of p-Aurora-A at the basal body of the primary cilium, reducing both the proportion of cells forming primary cilia and their length (Pejskova, et al. 2020). Since these changes mirror those observed in ZO-2 KD cells, and considering the interaction between ZO-2 and KIF14 (Capalbo, et al. 2019, Cho, et al. 2022, Couzens, et al. 2013, Go, et al. 2021), we next analyzed the expression of KIF14 in the cilia of parental and ZO-2 KD cells. Figure 7Ca-d shows expression of KIF14 at the base of procilia and along the axoneme of the primary cilia in parental cells. In contrast, a decreased expression of KIF14 is observed at the base of procilia of ZO-2 KD cells compared to parental cells (Fig. 7a,b,c,e,f). The quantification of KIF14 fluorescent intensity at the base of procilia in parental and ZO-2 KD cells confirms the decreased expression of the protein in ZO-2-depleted cells (Fig. 7Cg).

Our results suggest that ZO-2 acts as a scaffold at the basal body of the primary cilium, supporting the attachment of CEP164, KIF14, and IFT-B complex protein IFT57, which are crucial for the development of the primary cilium.

### Activation of the PI3-kinase/AKT signaling pathway contributes to the cilia loss observed in ZO-2-depleted cells

**Fig. 8:**
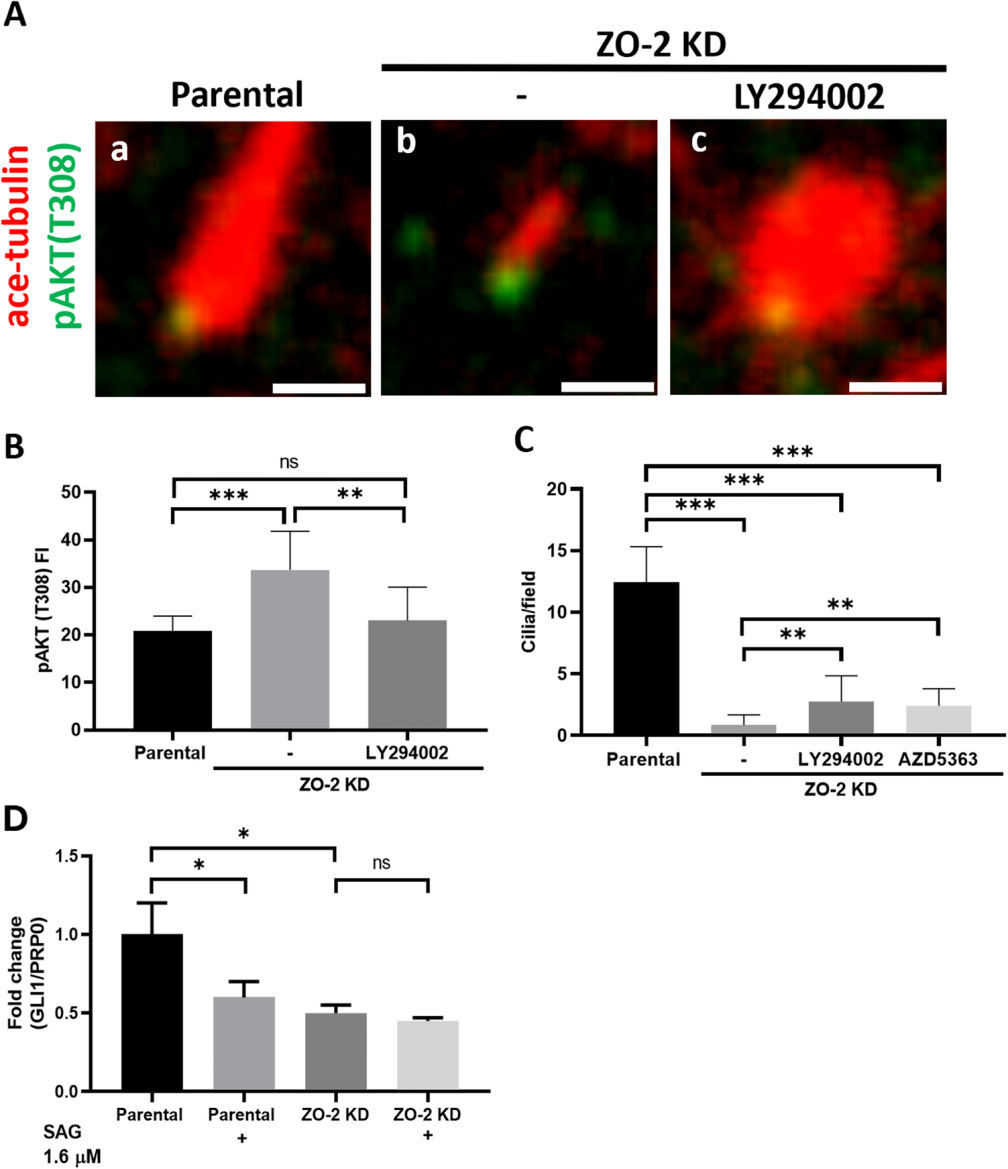
In ZO-2 KD cells, pAKT accumulates at the base of procilia, and SHH-dependent transcription is impaired. Parental and ZO-2 KD MDCK cells were plated to confluence and incubated in CDMEM for 24 h. The monolayers were then transferred to DMEM without serum for 24 h to stimulate cilium development, with or without 50 μM LY294002, a PI3-kinase inhibitor, or 3 μM of the AKT inhibitor AZD5363. **A** Representative immunofluorescence images obtained with antibodies against pAKT(T308) to detect active AKT and anti-acetylated tubulin to visualize cilia and procilia. Bars, 1 μm. **B** pAKT(T308) immunofluorescence intensity quantification at the ciliary base. 25 cilia were analyzed per experimental condition. Statistical analysis was done using the Mann–Whitney test, **p < 0.01, ***p < 0.001. **C** Quantification of cilia/field. 12 optical fields were examined per condition. Statistical analysis was done using the Mann–Whitney test. **p < 0.01, ***p < 0.001. **D** The lack of ZO-2 impairs SHH-dependent transcription. A qRT-PCR of Gli1 mRNA was done in parental and ZO-2 KD cells incubated for 24 h in serum-free media with or without 1.6 μM SAG, the agonist of the SMO receptor. Results from two independent experiments done in triplicates. Statistical analysis was done using the One-way ANOVA test. *p < 0.05

Activating the PI3-kinase/AKT signaling pathway promotes cilia loss, while PI3-kinase inhibition or AKT knockdown prevents cilia disassembly [for review see, (Conduit and Vanhaesebroeck 2020)]. The kinase AKT is recruited to the plasma membrane by activating its pleckstrin homology (PH) domain with PIP3/PtdIns(3,4)P_2_ (Frech, et al. 1997). AKT is then fully activated through phosphorylation by PDK1 at T308 and mTORC2 at S473 (Sarbassov, et al. 2005). PtdIns (3,4,5)P3 and AKT localize to the base of the cilia, and upon deletion of the phosphatase INPP5E, which hydrolyzes PtdIns (3,4,5) P3, the fluorescent signal of PtdIns (3,4,5)P3 and phosphorylated AKT (pAKT T308) at the cilia base is amplified, while the percentage of cells with cilia diminishes (Conduit, et al. 2017). The phosphorylation of GSK3β by AKT at S9 inhibits its activity, and deletion of INPP5E leads to increased GSK3β phosphorylation along the ciliary axoneme, resulting in subsequent cilia loss (Conduit, et al. 2017). These findings prompted us to analyze the expression of pAKT (T308) in the cilia of parental and ZO-2 KD cells. Figure 8Aa,b and B) shows that in ZO-2 KD cells, compared to parental cells, there is an increased pAKT(T308) signal at the cilia base. Additionally, we observed that treating serum-starved ZO-2 KD cells for 48 h with 50 μM of the PI3-kinase inhibitor LY294002 diminishes the pAKT signal at the base of the cilia (Fig. 8 Ac and B). These treatments, as well as the administration of 3 μM of the ATP-competitive inhibitor of AKT, AZD5363, partially rescue cilia development (Fig. 8C). These results suggest that the increased PI3-kinase/AKT signaling at the cilia base contributes, in part, to the lack of cilia development observed in ZO-2 KD cells.

### ZO-2 depletion impairs transcriptional regulation by the SHH pathway

Primary cilia are essential for mechano- and chemo-sensory perception and control several intracellular signaling pathways, including Sonic Hedgehog (SHH). Therefore, we next analyzed the impact of ZO-2 depletion on the expression of *Gli1*, a target gene of the canonical SHH signaling pathway [for review, see (Niewiadomski, et al. 2019)]. For this purpose, a qRT-PCR of Gli1 mRNA was performed in parental and ZO-2 KD cells treated or untreated with SAG, the agonist of the G-protein coupled receptor protein smoothened (SMO). In the inactive state of the SHH pathway, the patched (Ptch) receptor inhibits SMO. However, when an SHH ligand binds to Ptch, the inhibition of SMO is relieved, and the SHH pathway becomes active [for review, see (Niewiadomski, et al. 2019)]. The activity of SMO affects the bifunctional Gli transcription factors, which are members of the Kruppel-like factors with highly conserved Zn finger DNA-binding domains. Three Gli factors are present in mammals: Gli1, Gli2, and Gli3. Gli1 acts as a transcriptional activator, whereas Gli2 and Gli3 have both activator and repressor functions. When SMO is activated, full-length Gli proteins undergo a series of posttranslational modifications in the primary cilium that ultimately allow them to translocate into the nucleus, where they regulate the expression of SHH target genes, including *Gli1* [for review, see (Niewiadomski, et al. 2019)].

Figure 8D shows that in parental MDCK cells, activating the SHH pathway with 1.6 µM SAG inhibits Gli1 expression, indicating the activation of the repressor function of Gli transcription factors. In ZO-2 KD cells, Gli1 expression is diminished compared to parental cells, and treatment with SAG has no effect. Thus, ZO-2 depletion impairs transcriptional regulation by the SHH pathway.

### ZO-2 is present in the spermatozoid tail

**Fig. 9:**
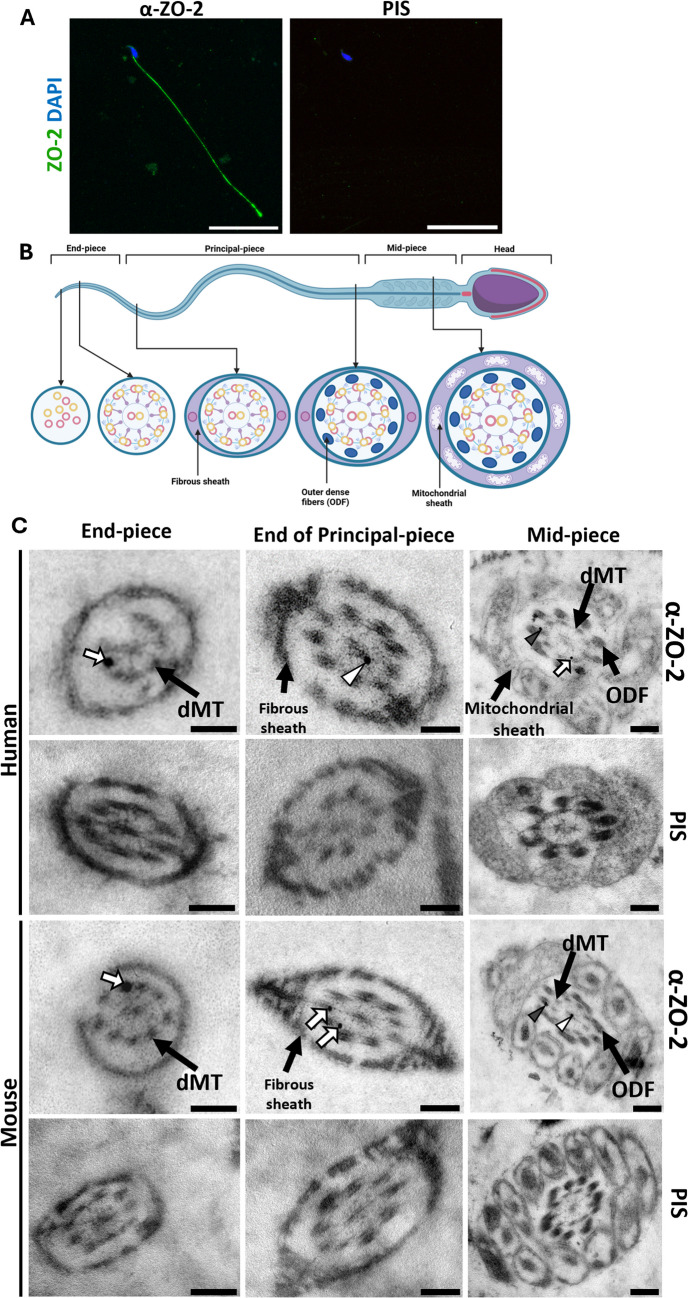
ZO-2 is present in the spermatozoid tail (**A**) Mouse spermatozoids were processed for immunofluorescence with rabbit antibodies against ZO-2 or rabbit PIS as negative control. Nuclei were stained with DAPI. Bars, 20 μm (**B**) Schematic illustration of the morphology of different sections of the spermatozoid tail. **C** Transmission electron microscopy images of mouse and human spermatozoids ultrathin sections incubated with rabbit antibodies against ZO-2 or rabbit PIS, followed by 20 nm gold-conjugated anti-rabbit antibodies. Bars, 200 nm. dMT, microtubule doublet; empty arrowhead, ZO-2 detected with gold conjugated antibodies colocalizing with the central microtubule doublet; full arrowhead, ZO-2 detected with gold conjugated antibodies colocalizing with ODF; empty arrow, ZO-2 detected with gold conjugated antibodies colocalizing with microtubule doublet

Since ZO-2 is found at the base of primary cilia, we conducted an analysis to determine its presence in mobile cilia as well. The localization of ZO-2 in mouse spermatozoa was examined using immunofluorescence and TEM in both mouse and human spermatozoa, employing antibodies against ZO-2 and rabbit pre-immune serum as a negative control. Figure 9A demonstrates that ZO-2 immunofluorescence staining occurs along the entire length of the mouse spermatozoon tail and is absent when rabbit pre-immune serum is used instead of the specific anti-ZO-2 antibody.

Figure 9B illustrates the structure of the spermatozoid tail, while Fig. 9C presents TEM images of spermatozoids treated with antibodies against ZO-2 or rabbit pre-immune serum, followed by secondary gold-conjugated antibodies. ZO-2 gold labeling appears in the mid-piece of mouse and human spermatozoids, seen in some of the nine dark spots corresponding to ODF2, within some of the nine pairs of microtubules, and in the central pair of microtubules. At the end of the principal piece of mouse and human spermatozoid tails, ZO-2 gold labeling associates with microtubule pairs at the periphery, as well as the central microtubule pair. Conversely, ZO-2 labels the peripheral microtubule pairs at the end of the tail. No gold labeling was observed in samples incubated with the rabbit pre-immune serum. These observations indicate that, unlike in primary cilia, where ZO-2 is found at the basal body, in spermatozoids, ZO-2 is distributed along the mobile tail, near ODF2 and microtubules.

## Discussion

Several studies have revealed the interaction and involvement of TJ proteins on centrosome and ciliary structure and function. Thus, the tricellular TJ proteins angulin-1, tricellulin, and ASPP2, as well as the bicellular TJ proteins occludin, claudin-7, ZO-1, and cingulin, colocalize by immunofluorescence microscopy in the centrosome with γ-tubulin (Konno, et al. 2020). Moreover, occludin regulates mitotic entry (Runkle, et al. 2011), while Par-3, a member of the Par-3/Par-6/Cdc42/aPKC complex that regulates TJ formation, colocalizes with γ-tubulin in the centrosome (Konno, et al. 2020) and is critical for the biogenesis of the primary cilium (Sfakianos, et al. 2007). In particular, proximity-dependent biotinylation assays done to discover proteins that interact with the centrosome and cilium (Gupta, et al. 2015) found that ZO-2 associated with the SDA proteins centriolin, Cep128, and cenexin/ODF2, as well as the cartwheel protein Cep135 (Ganapathi Sankaran, et al. 2019). These observations prompted the analysis of the location and function of ZO-2 in the centrosome.

Here, we found that ZO-2 is present in the centrioles, in the pericentriolar material of interphase centrosomes, and at the mitotic spindle poles. At the centrosomes, ZO-2 concentrates in the DA of the MC, colocalizing with CEP164 and only partially colocalizing with centriolin, an SDA protein, and the cartwheel component CEP135. ZO-2 does not appear to serve a structural function at the centrioles, as TEM images of centrioles in ZO-2-depleted cells are like those in parental cells. Instead, since ZO-2 is a scaffold protein to which multiple proteins associate [(for review, see (Gonzalez-Mariscal, et al. 2017)], it might play a role in the delivery or attachment of centriolar proteins. This proposal is consistent with the observation that in ZO-2-depleted cells, the immunofluorescence intensity at the centriole of CEP164 and CEP135 decreases, while that of centriolin increases.

That the absence of ZO-2, which concentrates in the DA, impacts the expression of the SDA protein centriolin, or the cartwheel protein CEP135, is not surprising given that there is a partial co-localization of ZO-2 with these proteins, and since the presence of elements of the DA influences the positioning of proteins in the SDA (Chong, et al. 2020).

Since the centrosome is the primary microtubule-organizing center, we analyzed the state of microtubules in migrating cells. We observed a reduction in acetylated microtubule arrays in wound-edge migrating cells lacking ZO-2, which could be restored upon re-expression of ZO-2. This finding is noteworthy because CEP164-depleted cells exhibit a reduction in stable, cold-resistant microtubules (Pitaval, et al. 2017), and ZO-2 KD cells have decreased expression of CEP164.

Tests were conducted to determine whether the absence of ZO-2 affected the development of astral microtubules and the mitotic spindle. The mitotic spindle consists of microtubules and various motor and microtubule-associated proteins [for review, see (Yount, et al. 2015)]. It was observed that ZO-2 depletion inhibits the interaction of EB1, which associates with the plus end of active polymerizing microtubules, with both the mitotic spindle and the astral microtubules radiating from the spindle poles. This observation indicates that ZO-2 favors the stability of astral and mitotic spindle microtubules. Furthermore, in ZO-2 KD cells, the levels of acetylated/stable tubulin at the spindle pole and in the mitotic spindle declined, confirming the importance of ZO-2 for microtubule stability.

To better understand the reduction in spindle length detected in ZO-2 KD cells, we analyzed NuMA expression. NuMA targets the minus-end of microtubules in the spindle poles, forming a complex with dynein and dynactin [for review, see (Kiyomitsu and Boerner 2021)]. The accumulation of NuMA induces liquid–liquid phase separation, triggering the formation of NuMA condensates that localize Kif2A at the spindle poles. Since Kif2A promotes poleward spindle microtubule flux, overexpression of NuMA has been linked to a reduction in spindle length (Sun, et al. 2021). In ZO-2 KD cells, we observed an increase in NuMA condensates at the spindle poles, which treatment with 1,6-hexanediol can disrupt, indicating that their formation results from liquid–liquid phase separation. Thus, the increase in NuMA condensates at the spindle poles in ZO-2 KD cells suggests an increase in poleward spindle microtubule flux, which would decrease mitotic spindle length. Additionally, mass spectrometry analysis has shown that NuMA interacts with the kinesin KIF14 (Sun, et al. 2021). Silencing KIF14 generates shorter and thinner mitotic spindles (Cai, et al. 2009) and abnormal chromosomal congression during mitosis (Zhu, et al. 2005). In ZO-2-depleted cells, we observed a reduction in the accumulation of KIF14 at the spindle poles. KIF14 co-immunoprecipitated with ZO-2, consistent with previous affinity binding assays (Capalbo, et al. 2019, Cho, et al. 2022, Couzens, et al. 2013, Go, et al. 2021) suggesting that ZO-2 may serve as a platform that concentrates KIF14 at the spindle poles, allowing it to modulate mitotic spindle organization and proper chromosomal congression.

In ZO-2 KD cells, the reduced length of the mitotic spindle correlates with a decreased accumulation of active Aurora at the spindle poles. This result aligns with previous observations indicating a decreased spindle length following Aurora-A inhibition (Sun, et al. 2021). The decreased expression of TPX2 at the spindle poles of ZO-2 KD cells may account for the reduced concentration of p-Aurora at these poles, which is expected to lead to the observed accumulation of NuMA at the spindle poles. This accumulation may contribute to the spindle misorientation previously reported in ZO-2 KD cells (Raya-Sandino, et al. 2017). These findings align with earlier studies indicating that when TPX2 lacks the Aurora-A binding region, NuMA becomes enriched at the spindle poles of mitotic cells and is no longer detected at the cell cortex, resulting in spindle misorientation (Polverino, et al. 2021).

The abnormal chromosome congression observed in ZO-2 KD cells during mitosis (Fig. 4Bb, Ed,e) may also originate from a reduced phosphorylation of NuMA at S1969 by Aurora-A, as seen in cells expressing the NuMA mutant S1969A (Sun, et al. 2021).

In the absence of ZO-2, the concentration of the proteins forming the NuMA/Aurora-A/TPX2 complex at the mitotic spindle poles is altered. As ZO-2 is a scaffold protein that associates with a wide variety of proteins [(for review, see (Gonzalez-Mariscal, et al. 2017)], it raises the possibility of ZO-2 playing a scaffolding role in the formation of the NuMA/Aurora-A/TPX2 complex at the spindle poles. Our results support this suggestion, as NuMA and Aurora-A co-immunoprecipitated with ZO-2, and were present in a ZO-2 pull-down. We did not detect TPX2 in the ZO-2 immunoprecipitate or the ZO-2 pull-down assay. However, a proximity-dependent biotin identification assay had previously demonstrated the interaction of ZO-2 with TPX2 (Huttlin, et al. 2021).

Our results also indicate that ZO-2 plays a role in regulating microtubule stability and function, as ZO-2-depleted cells exhibit a lack of cilia, decreased microtubule acetylation, and a mitotic spindle with reduced microtubule density. Previous observations have shown that TPX2 overexpression increases microtubule stability (Polverino, et al. 2021). Therefore, the reduced stability of microtubules found here in the mitotic spindle of ZO-2-depleted cells may also be a consequence of the reduced concentration of TPX2 at the spindle poles.

Other TJ proteins are known to organize microtubules; for example, cingulin interacts with microtubules (Yano, et al. 2013), while paracingulin recruits the microtubule minus-end to TJs through its binding to CAMSAP3, a microtubule minus-end-binding protein (Flinois, et al. 2024). The association of the microtubule-binding protein LUZP1 with TJs promotes apical constriction in epithelial cells (Yano, et al. 2021). We also found that ZO-2 depletion disrupted the organization of microtubules at the cell border, and previous observations in paracingulin KO mice revealed a disorganized microtubule architecture in the intestinal epithelium. These results suggest that various TJ proteins interact with microtubules and microtubule-binding proteins, thereby contributing to the organization of microtubules within the tissue.

The presence of ZO-2 at the basal body is crucial for cilia enlargement, as a delay in procilia development and the absence of fully elongated cilia were observed in cells lacking ZO-2, even when TEM detected no morphological differences between the basal bodies of parental and ZO-2 KD cells. We found that ZO-2 depletion resulted in a decrease in CEP164 at the basal body. CEP164 is indispensable for primary cilia formation (Graser, et al. 2007), as it mediates vesicular docking to the MC, a critical step in early ciliogenesis (Schmidt, et al. 2012). The MC's transition to the basal body involves the removal of CP110 and CEP97 from the distal end of the centriole (Spektor, et al. 2007) and the recruitment of intraflagellar transport (IFT) proteins. These processes require the activity of Tau tubulin kinase 2 (TTBK2) (Goetz, et al. 2012; Huang, et al. 2018), which is recruited to the basal body by CEP164 (Cajanek and Nigg 2014; oda, et al. 2014).

In ZO-2-depleted cells, we also observed the accumulation of p-Aurora at the basal body, which can activate HDAC (Pugacheva, et al. 2007), obstructing cilia formation due to tubulin deacetylation (Hubbert, et al. 2002). However, the inhibition of HDAC6 with tubastatin-A was insufficient to restore cilium formation in ZO-2-depleted cells, suggesting that other mechanisms also contribute to the inhibition of cilia formation in these cells. Since PtdIns (3,4,5)P3 and AKT localize to the cilia base, and the activation of the PI3-kinase/AKT signaling pathway promotes cilia loss [for review see, (Conduit and Vanhaesebroeck 2020)], we explored pAKT(T308) expression at the cilia base. We found that ZO-2-depleted cells exhibit an increased pAKT(T308) signal and a partial recovery in cilia formation in monolayers treated with PI3K or AKT inhibitors. This indicates that the accumulation of both Aurora-A and pAKT at the ciliary base contributes to cilia loss in ZO-2-depleted cells.

At the ciliary basal body, ZO-2 depletion reduces the concentrations of IFT57 and KIF14, suggesting that ZO-2 functions as a scaffold that aids in the recruitment of these proteins. In this context, several studies have demonstrated that ZO-2 associates with IFT57 (Go, et al. 2021) and with kinesin KIF14 (Capalbo, et al. 2019, Cho, et al., 2022, Couzens, et al., 2013), which we have also found to co-immunoprecipitate with ZO-2. IFT57 in photoreceptors is essential for ciliary elongation and cilia maintenance (Krock and Perkins 2008). IFT57 KO in renal and retinal pigment epithelial cells disrupts primary cilia development, and fibroblasts derived from a patient with defective IFT57, causing Bardet-Biedl syndrome, exhibited fewer ciliated cells, more abnormal cilia, and impaired anterograde transport in primary cilia (Nitoiu, et al. 2025). These observations imply that the reduced accumulation of IFT57 in the ciliary base could also contribute to the lack of cilia development in ZO-2-depleted cells.

KIF14 is a molecular motor that binds to microtubules and is essential for primary cilium formation and signaling through the SHH pathway (Brown, et al. 2015), indicating that its reduced expression in the basal body of ZO-2-depleted cells also contributes to the observed loss of cilia and the absence of response to SAG stimulation.

*Gli1* is a target gene of the canonical SHH pathway, and this route represents a *bona fide* ciliary signaling system (Niewiadomski, et al. 2019). Treatment of parental cells with SAG, an agonist of the SMO receptor, reduced the expression of *Gli1* mRNA as measured by qRT-PCR, indicating that in quiescent MDCK cells, SAG treatment activates Gli transcription factors with repressor functions. This transcriptional repression was not observed in ZO-2 KD cells, indicating a loss of SHH-dependent transcriptional regulation, consistent with the loss of cilia development. CEP164 interacts with the Gli2 transcription factor (Fushimi, et al. 2023), which is known to exhibit both activator and repressor functions (Niewiadomski, et al. 2019). CEP164 is essential for this factor's localization in the MC's DA, and depletion of CEP164 diminishes centriolar Gli2 localization (Fushimi, et al. 2023). Therefore, since the absence of ZO-2 reduces CEP164 concentration in the centriole and basal body, it may also negatively impact the centriolar recruitment of Gli2, thereby contributing to the lack of response of ZO-2 KD cells to SAG stimulation observed in this study.

Immunofluorescence revealed that ZO-2 is present along the tails of both mouse and human spermatozoa. These findings align with previous results indicating the presence of ZO-2 in mouse condensed spermatids (ortega-Olvera, et al. 2018). Using TEM and antibodies against ZO-2, in conjunction with secondary gold-conjugated antibodies, we detected ZO-2 labeling in the spermatozoa tail associated with microtubules and ODF2. This result agrees with a previous proximity-dependent biotinylation assay, which revealed the interaction of ZO-2 with ODF2/cenexin (Gupta, et al. 2015). ODF2 plays a crucial role in forming the outer dense fibers of the sperm tail, specifically in the mid and principal pieces. Mice exhibiting a high percentage of ODF2 chimerism are infertile and possess spermatozoa with tail defects, such as the absence of axonemal microtubule doublets, bent tails, and abnormal motility (Tarnasky, et al. 2010). It is also noteworthy that chimeric ZO-2 KO mice have a compromised blood-testis barrier and reduced fertility (Xu, et al. 2009). Furthermore, ODF2 is essential for forming the DAs and SDAs in the MC and generating primary cilia (Ishikawa, et al. 2005). In multiciliated epithelial cells of the trachea in mice, disruption of ODF2 exons leads to altered basal bodies, disrupting coordinated ciliary beating and resulting in a coughing or sneezing-like phenotype (Kunimoto, et al. 2012).

**Fig. 10:**
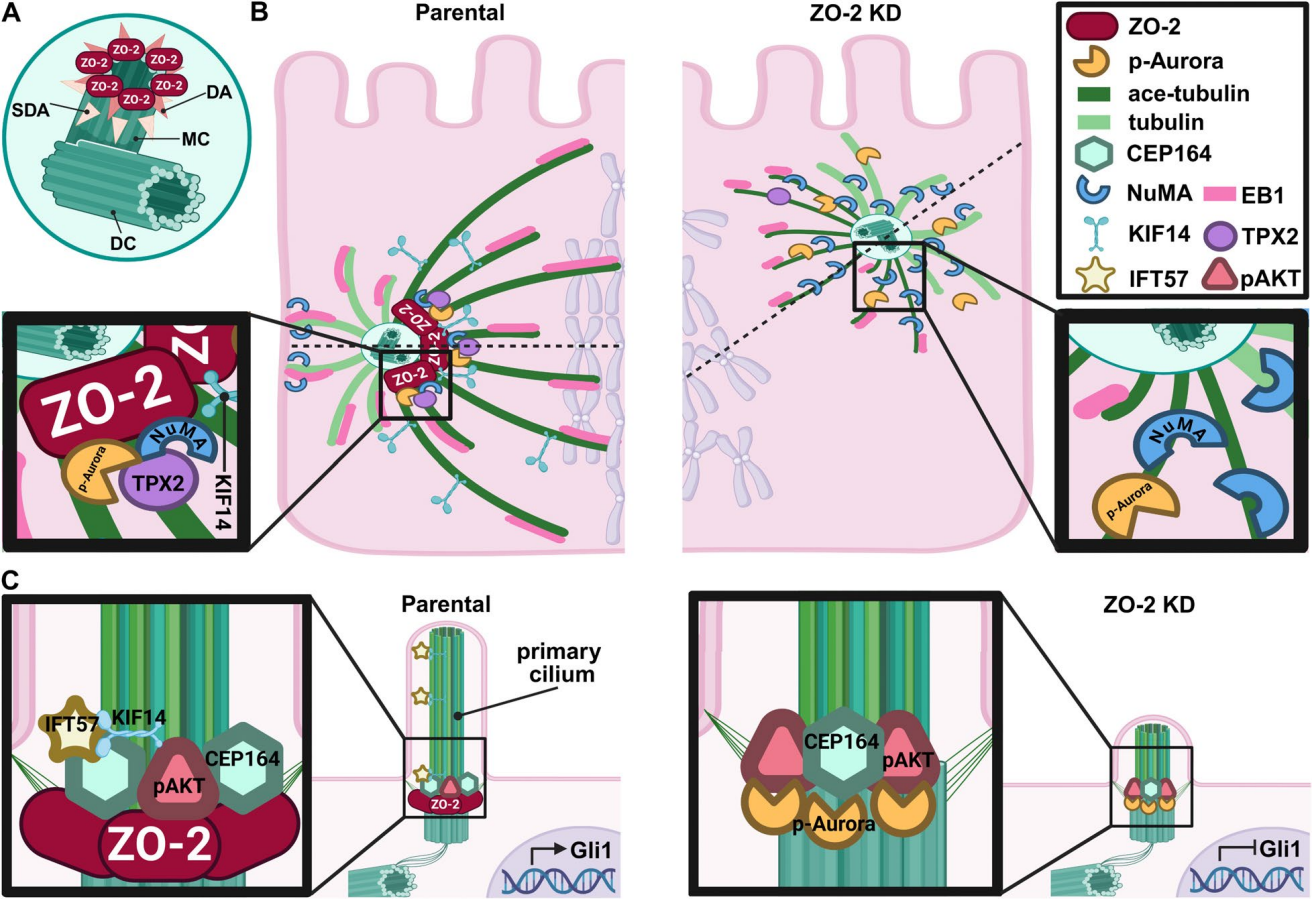
Schematic representation of the changes triggered in epithelial cells by ZO-2 depletion on the mitotic spindle and primary cilia. **A** ZO-2 is present at the distal appendages of the mother centriole. DA, distal appendage; SDA, subdistal appendage; MC, mother centriole; DC, daughter centriole. **B** ZO-2 depletion concentrates NuMA at the spindle pole and reduces the spindle pole accumulation of active Aurora (p-Aurora), TPX2, and KIF14. This suggests an increase in poleward spindle microtubule flux, which might explain the poor development of astral and mitotic spindle microtubules and the reduction in mitotic spindle length. The accumulation of NuMA at the spindle pole may also explain the previously reported spindle pole misorientation in ZO-2 KD cells (Raya-Sandino, et al. 2017), as illustrated by the dashed line. **C** In parental cells, ZO-2 functions as a scaffold at the ciliary basal body that promotes the concentration of CEP164, IFT57, and KIF14. Instead, ZO-2 depletion stabilizes the presence of p-Aurora and p-AKT at the basal body and reduces the accumulation of CEP164. Consequently, the development of primary cilia and Gli1 signaling through the SHH pathway is impaired

In summary, ZO-2 colocalizes with CEP164 in the DA of the MC and is present at the basal body of primary cilia, as well as in the tails of human and mouse spermatozoa. The absence of ZO-2 altered the cellular content of centriolar proteins CEP164, centriolin, and CEP135, but did not change the morphology of centrioles. Without ZO-2, p-Aurora and pAKT accumulated in the ciliary basal body, while CEP164, IFT57, and KIF14 concentrations diminished, inhibiting the development of primary cilia. ZO-2 is also found at the mitotic spindle poles, where it appears to act as a scaffold for KIF14 and the NuMA/Aurora-A/TPX2 complex. The altered accumulation of proteins at the spindle pole, triggered by ZO-2 depletion, may increase the poleward spindle microtubule flux. This, coupled with the observed microtubule instability, results in a shorter mitotic spindle. Collectively, these results indicate that, rather than being a centrosomal architectural component, ZO-2 enhances microtubule stability and functions as a platform for the correct accumulation of proteins at the spindle pole, the centriole, and the ciliary basal body, supporting the development of proper mitotic spindles and cilia (Fig. 10).

## Supplementary Information

Below is the link to the electronic supplementary material.

Supplementary file1 (TIF 27762 KB)

Supplementary file2 (TIF 14727 KB)

Supplementary file3 (TIF 21197 KB)

Supplementary file4 (TIF 109982 KB)

Supplementary file5 (TIF 17813 KB)

Supplementary file6 (TIF 16141 KB)

Supplementary file7 (TIF 48225 KB)

Supplementary file8 (TIF 16971 KB)

## Data Availability

No datasets were generated or analysed during the current study.
