# Supplementary figures and images for: ZO-2 is a scaffold at the centriole and mitotic spindle poles that enhances microtubule stability and supports the proper development of mitotic spindles and cilia
Source: Cell Tissue Res. 2025 Jul 29;402(1):21–50. doi: 10.1007/s00441-025-03992-0 (PMC12484294; doi:10.1007/s00441-025-03992-0)

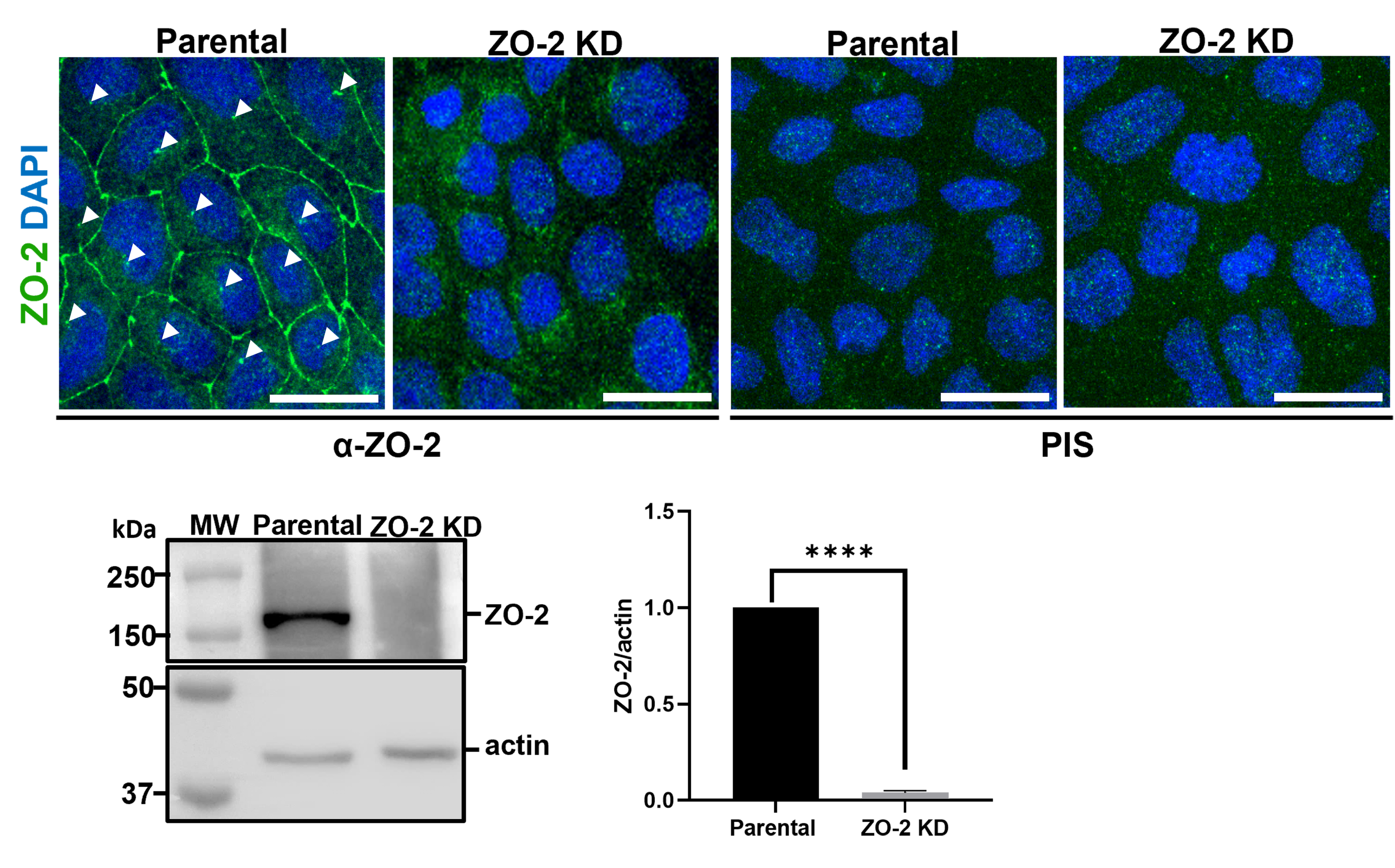

Supplement: Supplementary file 1 — Supplementary file1 (TIF 27762 KB) [file 441_2025_3992_MOESM1_ESM.tif]

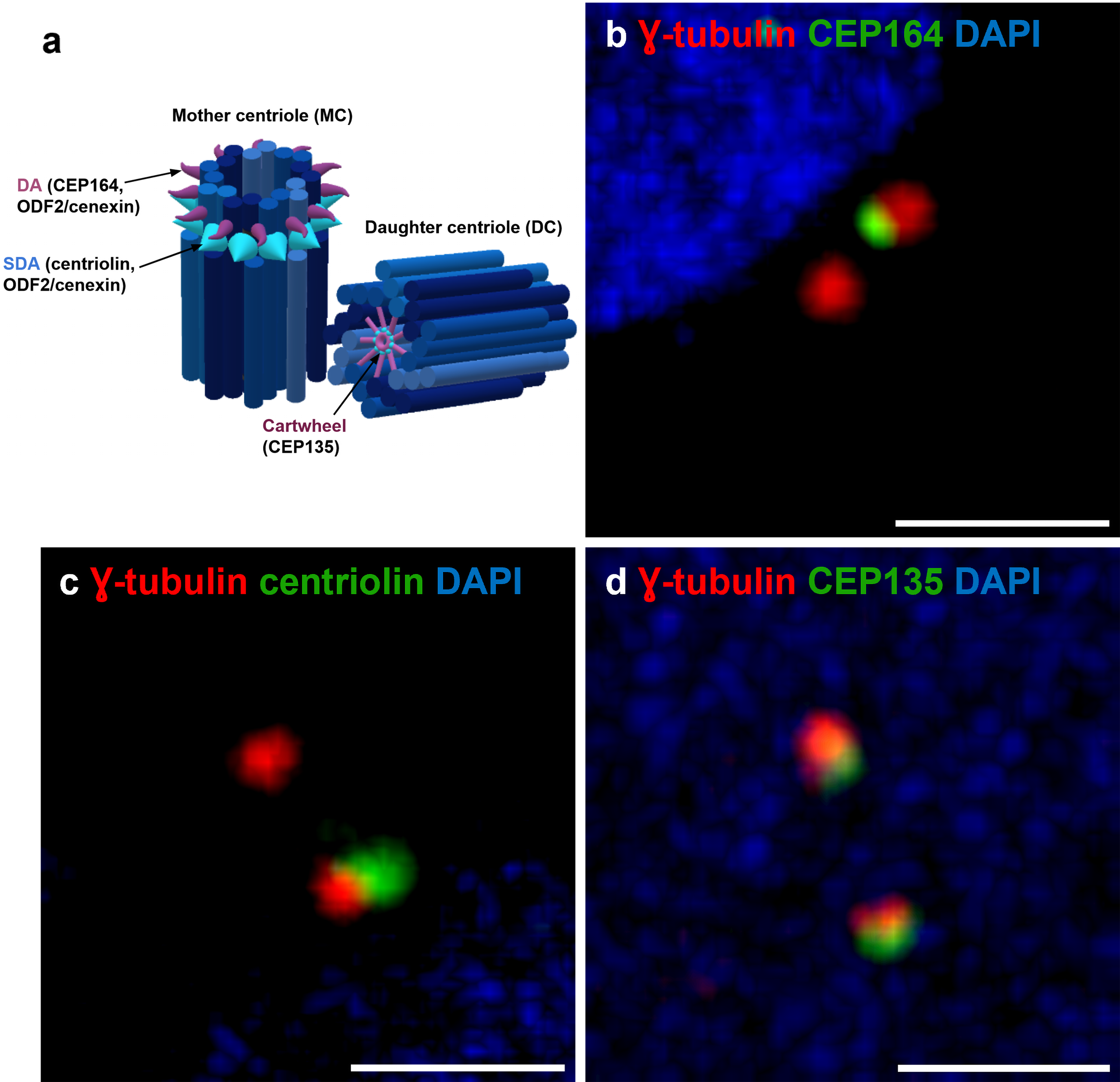

Supplement: Supplementary file 2 — Supplementary file2 (TIF 14727 KB) [file 441_2025_3992_MOESM2_ESM.tif]

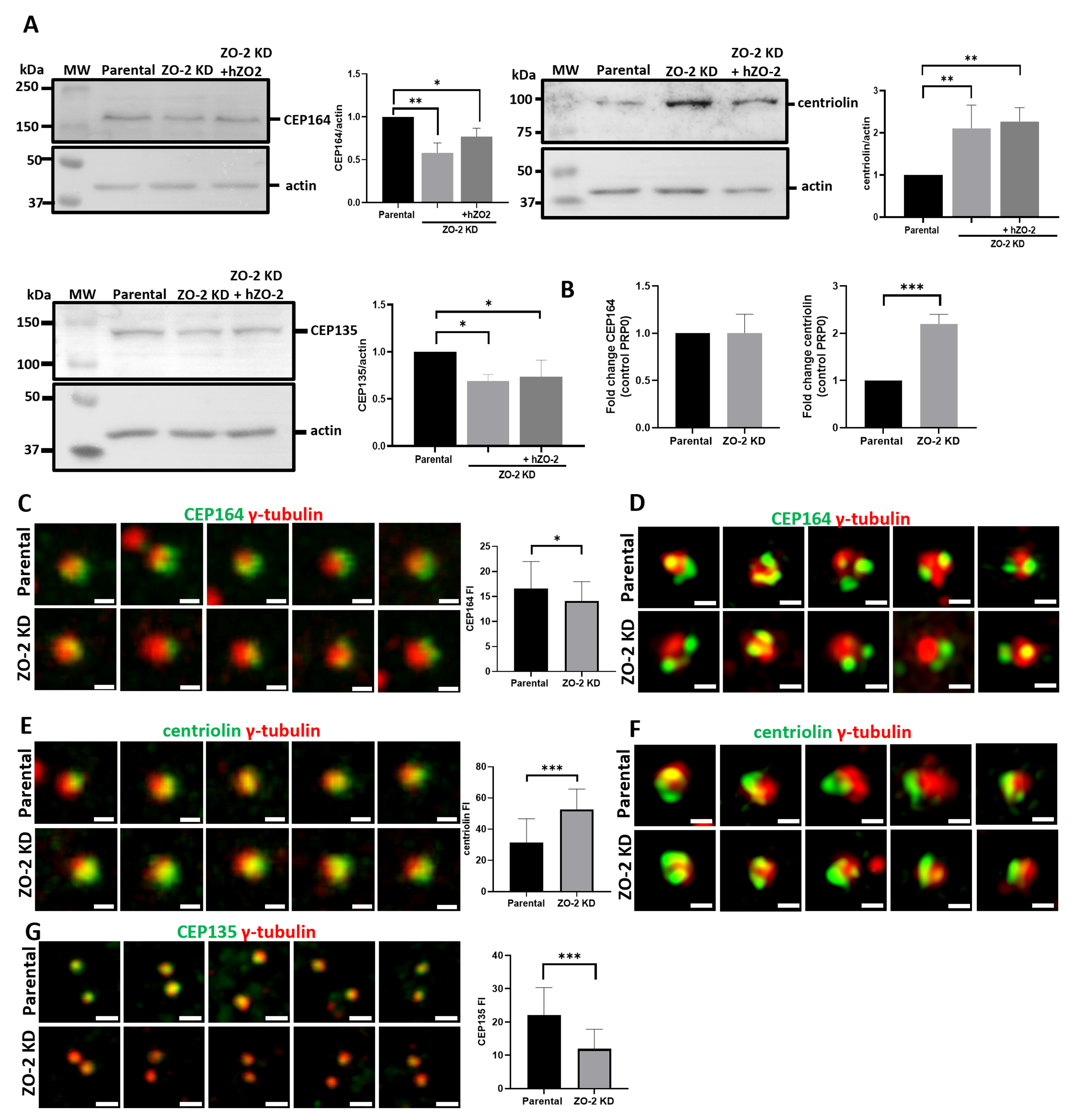

Supplement: Supplementary file 3 — Supplementary file3 (TIF 21197 KB) [file 441_2025_3992_MOESM3_ESM.tif]

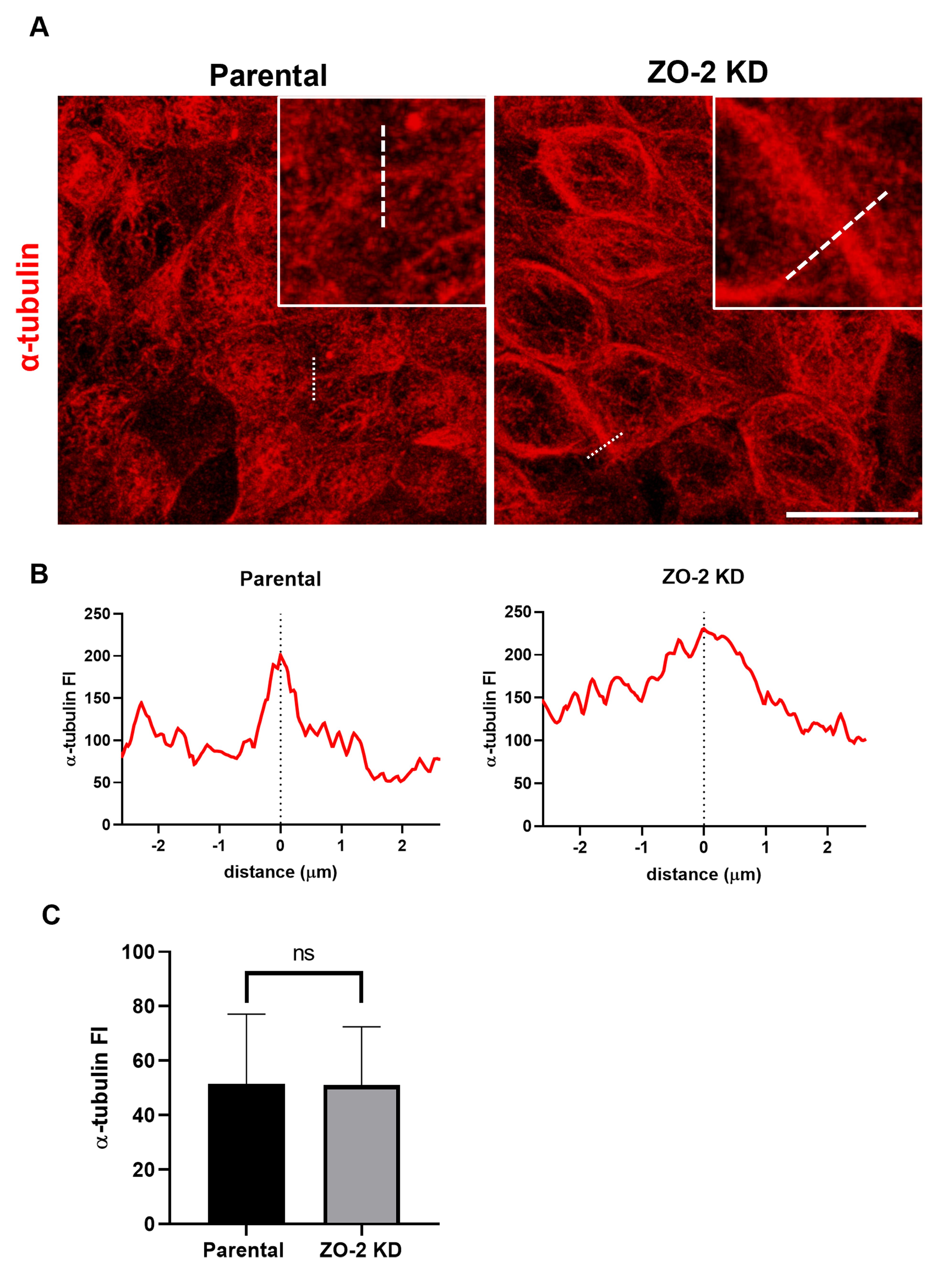

Supplement: Supplementary file 5 — Supplementary file5 (TIF 17813 KB) [file 441_2025_3992_MOESM5_ESM.tif]

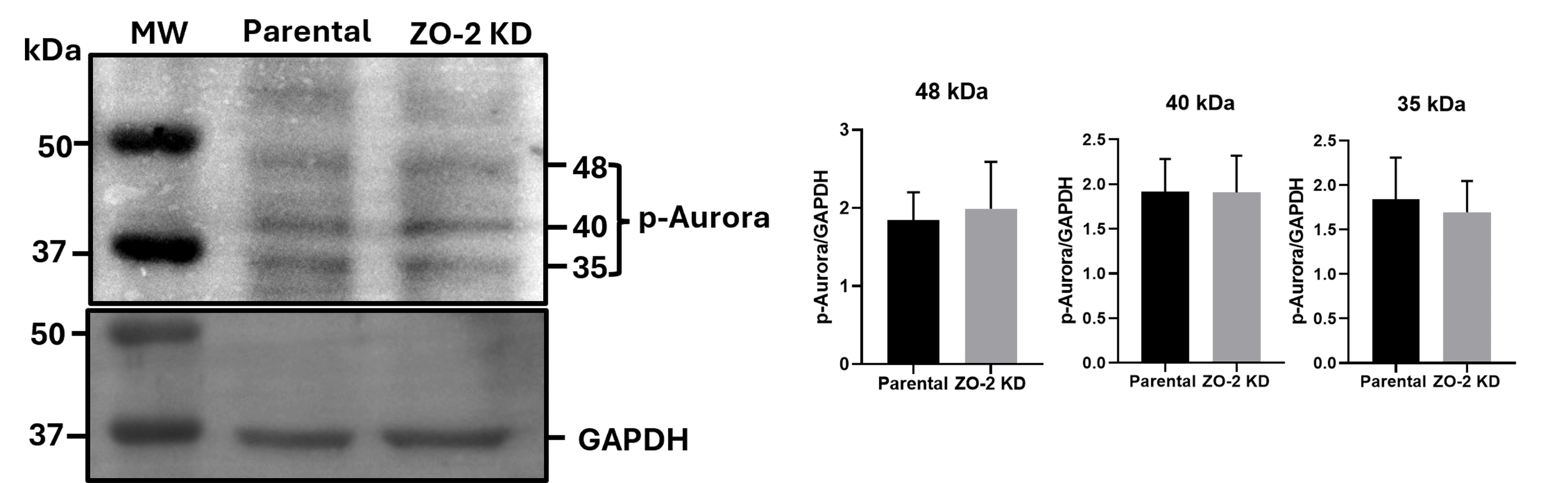

Supplement: Supplementary file 6 — Supplementary file6 (TIF 16141 KB) [file 441_2025_3992_MOESM6_ESM.tif]

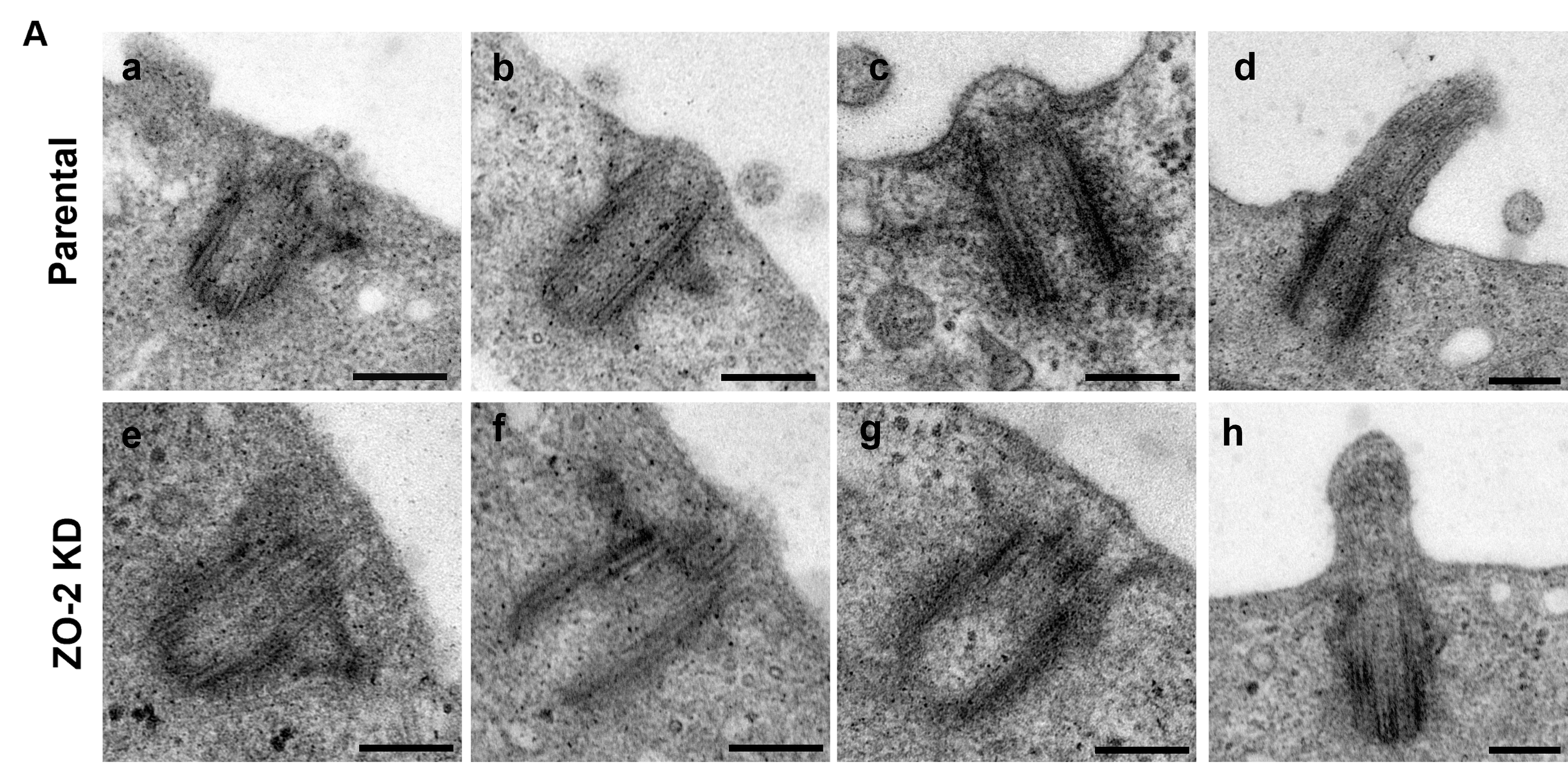

Supplement: Supplementary file 7 — Supplementary file7 (TIF 48225 KB) [file 441_2025_3992_MOESM7_ESM.tif]

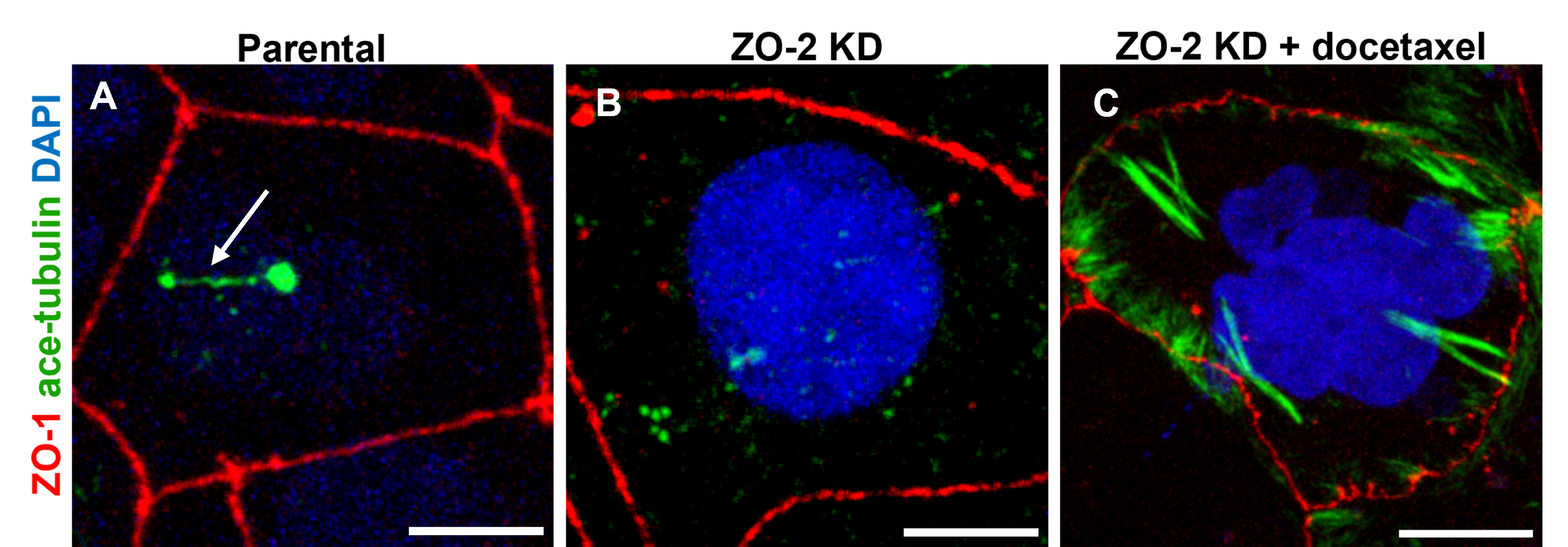

Supplement: Supplementary file 8 — Supplementary file8 (TIF 16971 KB) [file 441_2025_3992_MOESM8_ESM.tif]
